# Carbon Nanotubes, Graphene, and Carbon Dots as Electrochemical Biosensing Composites

**DOI:** 10.3390/molecules26216674

**Published:** 2021-11-04

**Authors:** Raja Ram Pandey, Charles C. Chusuei

**Affiliations:** Department of Chemistry, Middle Tennessee State University, Murfreesboro, TN 37132, USA; RajaRam.Pandey@mtsu.edu

**Keywords:** carbon nanotubes, graphene, carbon dots, metal oxide, nanoparticles, noble metal, surface functionalization

## Abstract

Carbon nanomaterials (CNMs) have been extensively used as electrochemical sensing composites due to their interesting chemical, electronic, and mechanical properties giving rise to increased performance. Due to these materials’ unknown long-term ecological fate, care must be given to make their use tractable. In this review, the design and use of carbon nanotubes (CNTs), graphene, and carbon dots (CDs) as electrochemical sensing electrocatalysts applied to the working electrode surface are surveyed for various biosensing applications. Graphene and CDs are readily biodegradable as compared to CNTs. Design elements for CNTs that carry over to graphene and CDs include Coulombic attraction of components and using O or N atoms that serve as tethering points for attaching electrocatalytically active nanoparticles (NPs) and/or other additives.

## 1. Introduction

Graphene-based carbon nanomaterials (CNMs), namely carbon nanotubes (CNTs; both single- and multi-walled), graphene and carbon dots (CDs), show wide-ranging applications in biosensors with greater lifetimes due to their cheaper production and promise of low toxic nature [1,2]. However, more recent studies have demonstrated cytotoxic effects of CNTs in various cell lines and animal models [3]. CNTs are not soluble in aqueous solution and can form colloidal suspensions, which persist in the environment. Even if CNMs are relegated to in vitro assays, concerns remain regarding their potential environmental impact [4]. Studies of CNTs and graphene in four aquatic systems in the southeastern United States indicate longer-term effects in a hypothetical 50-year CNM release simulation [5]. To date, no methods have been developed to render CNTs biodegradable. Strategies have been developed to biodegrade graphene and CDs. Kurapati et al. have shown that graphene can be biodegraded via *neutrophil myeloperoxidase*-mediated catalysis [6]. Biodegradation of CDs can be achieved using sequential enzyme oxidation [7]. Biosensing applications are essential for monitoring clinical drugs, food security, and diagnosis of various diseases [8].

In developing CNM-based electrochemical sensing electrode technology, there has been a natural progression in incorporating insights from CNTs to the more recent appearance of graphene and carbon dot materials. This review aims to provide the reader with a survey of this family of graphenal materials used as biosensing composites, noting the surface structural effects of electrocatalysts on sensing performance. Graphene in CNMs have a negative environmental impact due to its ability to bind toxic heavy metals and increase their transport via colloidal dispersion. While the current state of technology has not made all CNM forms readily biodegradable, insights can still be gained by recognizing structural feature correlations that can be exploited and made transferable to carbon dots for improved electrocatalyst design. If facile methods are developed to decompose CNTs and graphene, new design efforts would ultimately diversify biosensing technology.

Electrochemical techniques surveyed in this article include cyclic voltammetry (CV), chronoamperometry (CA), square wave voltammetry (SWAV), differential pulse voltammetry (DPV), electrochemical impedance (EIS), and electrochemical luminescence (ECL) to examine the efficacy of modifying the glassy carbon electrode surface. A common fabrication motif is the use of tethered O atom or N atom containing moieties that serve as effective tethering points for catalytically active additives to the CNTs composites. NPs can also be electrostatically attached to the carbon surface. These keystone structural design features in CNT composites are transferable to the more environmentally friendly graphene and CD supports and encapsulants.

## 2. Experimental and Discussion

**Scheme 1:**
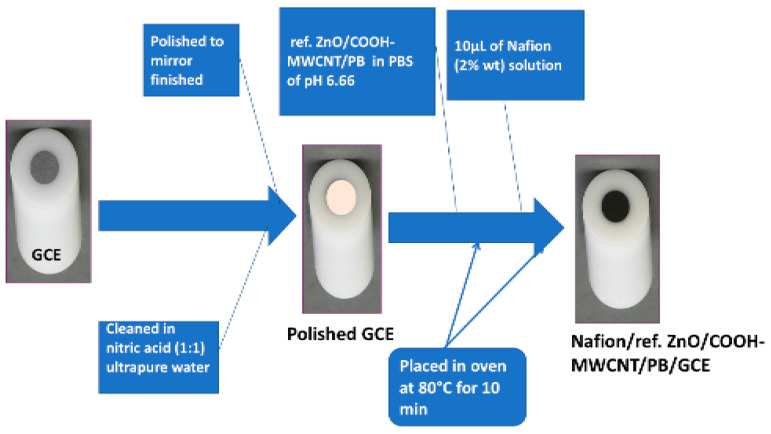
Depositing modified/synthesized (CNMs) onto the GCE surface.

The general scheme for modification of the glassy carbon electrode (GCE) as an electrochemical sensor is shown in Scheme 1. The activation of GCE by HNO_3_ is required before effectively depositing the electrochemical sensing CNMs. In this example, a 2 wt% Nafion is used to bind CNM electrode additives without interfering with working electrode performance.

### 2.1. Carbon Nanotube Composites

CNTs have high surface areas in which small molecules can be attached to their sidewalls via chemical functionalization. CNTs were first observed in 1952 [9]. CNTs, varying from 4 to 30 nm in diameter and up to 1 µm in length, were re-discovered by Iijima [10]. CNTs can be characterized in terms of variable diameter, length, the number of layers (cylindrical/tubular structures), and vectors of chirality. They are divided into two fundamental types: single-walled carbon nanotubes (SWCNTs) and multi-walled carbon nanotubes (MWCNTs) [11].

#### 2.1.1. Functionalizing CNTs

Commercialized CNTs with heavily entangled bundles exhibit inherent difficulties in dispersion [12]. To avoid such limitations, various methods have been developed to alter the surface morphology of CNTs, which are important for enhancing the deposition of metal nanoparticles in the fabrication of composite by providing tethering points for their attachment. Such functionalization matters in the context of chemical sensing because it helps to attach metal nanoparticles. The agglomeration of tethered nanoparticles can be minimized and thereby increase the surface area of the composite for greater sensitivity. Furthermore, functionalizing the CNT sidewalls is essential to prevent the agglomeration of nanoparticles (NPs) as O-containing functional groups provide tethering points to improve their dispersion.

Covalent bonding between the layers and small molecules can be carried out using covalent functionalization. General modification of CNTs occurs with small molecules as a pre-modification step. Covalent modification of CNTs with small molecules is required to increase dispersibility to functionalize them with the aim of fabricating biosensing electrocatalysts. The modification affects the compatibility of the matrix and fillers are affected [13,14,15,16,17,18,19,20]. Carbon paste (CPE) and carbon fiber (CFE) electrodes are made in this fashion. To synthesize various O-containing groups on the surface of CNTs, carboxyl, hydroxyl, and epoxy groups are created on the CNTs using oxidation reactions with aqueous H_2_O_2_ and concentrated sulfuric acid [21,22,23]. The degree of oxidation on CNTs is controllable using the ratio of sp^3^ to sp^2^ hybridized carbon atoms with the increase in the ratio of sp^3^ to sp^2^ carbon denoting greater oxidation on the surface of CNTs. Methods include condensation via amidation [24], esterification [25], acylation [26], and silanization reactions with small molecules [27]. The function of such moieties is to serve as tethering points for electrocatalytically active NPs. Oxidized CNTs can also undergo condensation with oxygen-containing functional groups using acid chlorides as intermediates [28] or through carbodiimide-activated coupling [29]. For electrochemical sensing applications, the formation of these functional groups is important for enhancing the surface area of nanocomposites, resulting in enhancing the sensitivity of biological analytes.

One example of attaching NPs to CNT sidewalls is by reducing silver ions to silver NPs; the NP-decorated organic functionalized MWCNTs are used to detect glucose electrochemically [30]. In this covalent functionalization method, MWCNTs were well dispersed in tetrahydrofuran (THF) and SOCl_2_ and treated with diethylenetriamine via an acylation reaction. MWCNT-CO-NH-cyanuric-NH_2_ and MWCNT-CO-NH-cyanuric as CNT composites were generated by acylation between CNT-CO-NH_2_ with cyanuric chloride and diethylenetriamine, respectively.

#### 2.1.2. Strategies to Modify the Working Electrode Surface

To increase the density of O-containing functional groups to the CNT sidewalls, sonicating the CNTs in the presence of nitric acid and sulfuric acid is commonly used. MWCNTs are functionalized to attach metal nanoparticles, thereby increasing the electroactive surface area, leading to greater electrochemical sensitivity [31]. The cavitation process of sonication is used in this manner to increase the density of -COOH and –CO- moieties on the MWCNT surface. As a result, finely dispersed NPs are attached to the MWCNT sidewalls. Sonication time can be varied to control the surface density of moieties and thereby achieve greater electrocatalytic activity [32]. Pt nanoparticles have successfully been attached to functionalized MWCNTs using this sonication procedure [33]. This sonication approach has been used to attach zinc oxide (ZnO) NPs to the COOH-functionalized MWCNT sidewalls, useful for uric-acid sensing [34]. Sonication was also used to combine CoO NPs with MWCNTs for assaying dopamine using CV [35].

Electrostatic attraction is another important strategy for attaching two different components based on differences in surface charge. The point of zero charge (PZC) is the pH at which the solid surface is electrically neutral under aqueous solution conditions depending on the ability of hydroxylated surfaces to become protonated or deprotonated on the solid-aqueous solution surface, according to electrical double layer theory [36,37]. The PZC of CNTs can be controlled by attaching electron-donating or withdrawing moieties to the sidewalls to deposit well-dispersed metal nanoparticles. Having components with different PZC values requires attachment at an intermediate pH value. Under these conditions, entities with a PZC below the solution pH will adopt a negative charge. Similarly, entities with a PZC above the solution pH will adopt a positive charge. These opposite charge adoptions of the two components serve as the driving force for attachment, forming the composite.

**Scheme 2:**
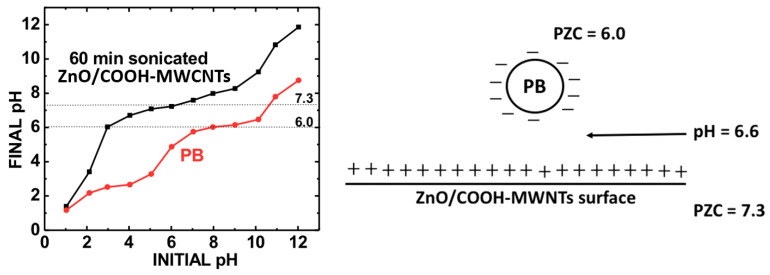
The determination of PZC of ZnO/MWCNTs and PB. Right: attaching PB with the composite, ZnO/MWCNTs using PZC (left). Reproduced with permission from ref. [39]. Copyright 2019, American Chemical Society.

CNT surfaces can adopt a wide range of PZCs existing through chemical functionalization [38]. The PZC of ZnO/MWCNTs and Prussian Blue (PB) were experimentally determined to be 7.3 and 6.0, as shown in Scheme 2, applying a procedure developed by Park and Regalbuto [37]. When such materials [ZnO/MWCNTs (PZC = 7.3) and PB (PZC = 6.0)] were stirred in PBS with pH 6.6, an intermediate pH condition that is between the PZCs of these materials, PB adopted an overall negative charge while ZnO/MWCNTs adopted a positive surface charge as shown in the scheme in Scheme 2 (right-hand drawing) [39]. In this process, the attachment of PB on the surface of ZnO/MWCNTs via Coulombic attraction was successfully accomplished. This action resulted in enhanced electro-composite sensitivity for the detection of H_2_O_2_, a biomarker for oxidative stress. Gatabi et al. also applied differing PZC values to attach maghemite NPs onto the surface of ox-MWCNTs (oxidized MWCNTs). The PZC of maghemite is 8 while that of ox-MWCNTs is 2.66. Decorating the MWCNTs with maghemite NPs was achieved in a slightly acidic medium [40]. Maghemite-MWCNT electrochemical composites are useful in assaying resorcinol, a widespread industrial waste product that is also an endocrine disruptor [41]. Selectivity depends not only on the specific potential obtained from CV, but also on the Coulombic interaction, i.e., either attractive or repulsive forces between the pK_a_ value of the analytes and the PZC of the composite. Therefore, the principle of selectivity via altering PZC is extended to the more complex composites. Deb et al. used differences in the PZC of a Pt/MWCNT composite with those of ascorbic acid, acetaminophen, and H_2_O_2_ to selectively detect them using CA (*vide infra*, Section 2.1.3) [42].

A final design aspect of the CNT electrode composites is the use of capping agents, which are needed for the working electrode surface to prevent the composites from dislodging from the working electrode. Nafion and Chitosan are commonly used binders to make the composite stable on the surface of glassy carbon electrodes [39,43]. Nafion is a common name for sulfonated tetrafluoroethylene-based fluoropolymer-copolymer. As a result of incorporating trifluorovinyl ether groups terminated with sulfonate onto a tetrafluoroethylene backbone, Nafion displays unique ionic properties. Nafion is a highly conductive material, and it prevents chemical alterations on the GCE surface. In fact, the sulfonate groups of Nafion prevent chemical attack via electrostatic repulsion and hydration effect [44,45]. This additive is required to avoid electro-composites from dislodging the GCE surface.

Nafion, with a 1–5 wt% solution concentration, is used to encapsulate the composite onto the working electrode surface. Similarly, chitosan is a natural polysaccharide synthesized from shrimp shells and characterized by abundant -OH and -NH_2_ groups. Chitosan and its composites have been widely used in drug and gene delivery, tissue engineering, and biosensing because of their high biocompatibility, biodegradability, hydrophilicity, mechanical strengths, antibacterial properties, and good processability in films. Chitosan enhances both the stability and lifetime of the sensor as it causes dispersion of the biological recognition element in a biomimetic environment [46].

#### 2.1.3. Biosensing Applications of CNT Composites

##### Noble Metal-Based Composites

**Scheme 3:**
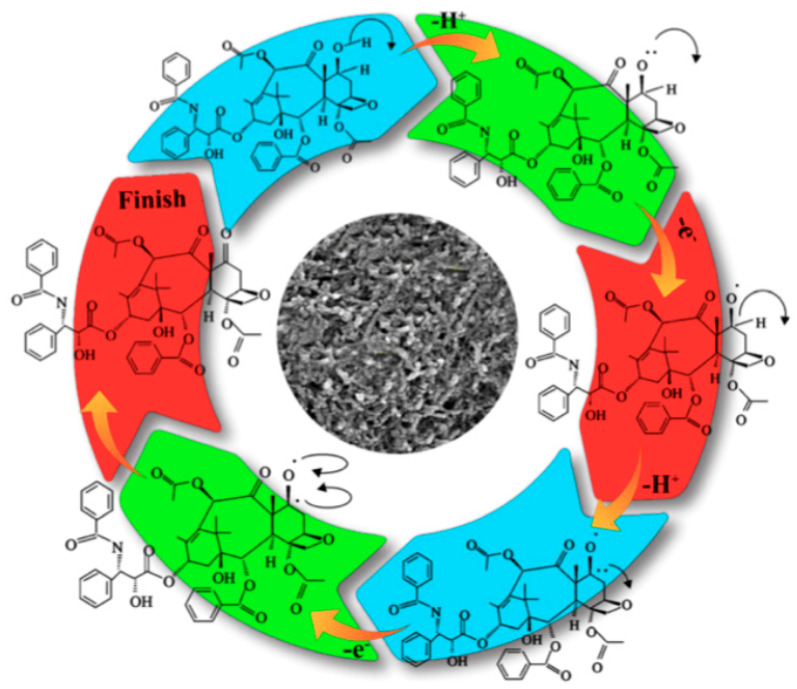
Electrooxidation mechanism of Dox at Au-MWCNTs/GCE. Reproduced with permission from ref. [48]. Copyright 2018, Springer Nature.

Docetaxel (Dox) is commonly used as an anticancer drug for treating lung, breast, prostate, ovarian, stomach, neck, and head cancer and is registered as the most effective and safe medicine in the world according to the World Health Organization [47,48]. However, monitoring exact levels of Dox in real samples (human urine and human serum) is required due to its various side effects. Najari et al. reported a selective and sensitive method for quantifying Dox at the Au-MWCNTs/GCE surface using CV and differential pulse anodic stripping voltammetry (DPASV) [48]. The introduction of MWCNTs on the GCE surface enhances electric conductivity. In addition, decorating MWCNTs with Au nanoparticles helps to improve the conductivity of MWCNTs-modified electrodes and signal response. This sensor can detect Dox in the range of 0.3–3.3 µmol∙L^−1^ with the LOD of 90 nmol∙L^−1^. Such a sensor is selective against Na^+^, K^+^, Cl^−^, Mg^2+^, Fe^3+^, CO^2−^, NO_3_^−^ ions along with glucose, sucrose, fructose, lactose, ascorbic acid, dopamine, thiourea, and alanine on the DPASV measurements. Only oxidation peaks were observed under the analysis of Dox at Au-MWCNTs/GCE surfaces. The number of electrons shifted during the electrooxidation of Dox on the sensor’s surface becomes equal to the number of protons. Such an electrooxidation mechanism for Dox at the surface of the sensor is shown in Scheme 3. The discharge of two electrons and protons was seen under this mechanism. Oxidation current response is proportional to the concentration of the analyte.

A nanocomposite of Pt/Ni(OH)_2_/MWCNT was synthesized using a simple two-step nitrite sensing method. This sensor showed enhanced electrocatalytic activity and excellent reliability. The nanocomposite was formed from synergistic effects between Pt NPs and Ni(OH)_2_/MWCNTs composites. In this composite, Ni(OH)_2_/MWCNTs offer a large surface area which helps to load the Pt NPs and improve the electronic conductivity. This noble nanomaterial-based nitrite sensor exhibits a low detection limit, good reproducibility, and excellent selectivity to quantify nitrite. This analyte is generally found in foods. Monitoring the concentration of nitrite is important to prevent food poisoning. An excess of it leads to irreversible hemoglobin oxidation in the human body, decreasing the blood oxygen-transporting capacity. In addition, nitrite reacts with amines to become carcinogenic nitrosamines, leading to conditions such as hypertension and gastrointestinal cancer [49].

Delerue-Matos et al. studied screen-printed carbon electrodes (SPCEs)-MWCNTs/Au NPs and SPCE-Au NPs to improve the electrochemical analysis of the extracellular domain of the human epidermal growth factor receptor 2 (HER2-ECD) using linear sweep voltammetry [50]. In this analysis, the combination of MWCNT with Au NPs had the highest sensitivity. The antibody concentrations were optimized and applied to develop biosensing strategies on SPCE-AuNP and SPCE-MWCNT/Au NP with LODs of 8.5 ng/mL and 0.16 ng/mL, respectively, well below the cut off value of 15 ng/mL for the breast cancer biomarker. Also, the linear dynamic range of this sensor was reported between 7.5 ng/mL to 50 ng/mL.

Cheng et al. described that MWCNTs/poly(3,4-ethylenedioxythiophene(PEDT))-gold nanocomposites were readily fabricated using a one-pot method [51]. In this process, Au-nanoparticles were synthesized by reducing hydrogen tetrachloroaurate with a 3,4-ethylenedioxythiophene (EDT) monomer, which was simultaneously oxidized and polymerized. The modified sensor prepared from this composite exhibits superior electrochemical sensing performance for luteolin detection using EIS, CV, and SWV. This fabricated luteolin sensor shows good selectivity, stability, and reproducibility. The outcomes indicate that the excellent electrochemical performance of the electrodes with MWCNTs/PEDT-Au nanocomposites are assigned to the synergistic catalytic effect of the high specific area of MWCNTs and catalytically active sites of PEDT-Au. This sensor is used to quantify luteolin (3,4,5,7-tetrahydroxy-flavone), a natural flavonoid compound. Luteolin is abundant in fruits, seed shells, and vegetables, and it has been widely used in clinical applications for the treatments of some serious coronary heart diseases. Some toxic side effects are observed at high doses greater than 20 µM concentrations.

Carboxylic acid-functionalized carbon-allotropic nanomaterials (GO-COOH/MWCNTs-COOH) tethered to gold nanoparticles (Au NPs) have been fabricated for biosensing of 8-hydroxy-2′-deoxyguanine (8-OHdG). The composite sensing materials consist of GO-COOH, MWCNTs-COOH, and polyethyleneimine (PEI). In this composite, Au NPs were synthesized by self-assembly of negatively charged Au NPs onto positively charged PEI-wrapped GO-COOH and MWCNTs-COOH via electrostatic interactions. This composite was generated by simple electrostatic adsorption under sonication and centrifugation. This sensor shows high electrochemical performance for the oxidation of 8-OHdG, a biomarker of oxidative DNA damage. The analysis of this molecule is important for the diagnosis and monitoring of various diseases related to oxidative stress, using differential pulse voltammetry (DPV) [52]. This composite is amenable to urinalysis as it avoids signal interference from uric acid with the assistance of uricase. UA is oxidized to electrochemically inactive allantoin by uricase. The addition of uricase eliminates the interference of uric acid for the quantification of 8-OHdG. The UA peak diminished with the addition of 50 µg∙mL uricase, enhancing the sensor’s selectivity for 8-OHdG. The results of analytical detection of 8-OHdG in human urine samples, trace content, and complex matrices were promising, with a very high recovery percentage (at least 99.43%).

The selectivity of analytes depends on the specific voltage in which the voltages for reduction and oxidation for various analytes are well separated from one another. The measured PZC of the Pt-DEN-PANI-CNT composite was observed to be 6.8 experimentally [42]. Under pH 7.0 conditions, the surface charge of the Pt-DEN-PANI-CNT electrode adopts a negative charge due to a higher degree of surface hydroxylation than protonation. The composite is Coulombically attracted to H_2_O_2_ and APAP since their isoelectric points (denoted by their pK_a_ values) are at 11.75 and 9.38, respectively. Such analytes exist in their protonated forms at pH 7.0. On the other hand, ascorbic acid (AA) has an isoelectric point of 4.2 and therefore exists in a deprotonated state at pH 7.0. The deprotonated AA is negatively charged owing to 4 hydroxyl groups within its molecular makeup. AA would be repelled from the Pt-DEN-PANI-CNT surface. Coulombic attraction between composite and APAP and H_2_O_2_ served as the driving force for the electrocatalyst’s selectivity to these analytes not present with AA.

**Scheme 4:**
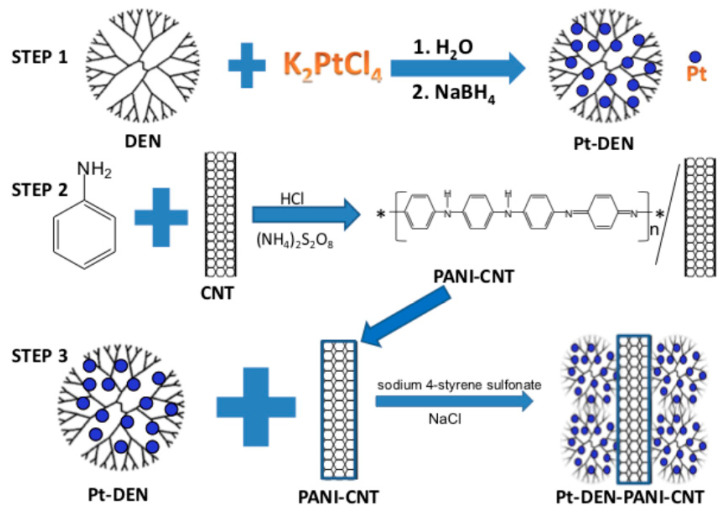
Fabrication steps to produce the Pt-DEN-PANI-CNT composite. Reproduced with permission from ref. [42]. Copyright 2016, Springer Nature.

The strategy behind this electro-composite fabrication differs in that for SWCNTs. The diameter is too small to incorporate tethered moieties for the much larger electroactive NPs; therefore, another approach is used. Instead, N atoms are used as tethering points to attach dendrimers, which encapsulate the electroactive NPs. The synthesis of nanocomposite is summarized in Scheme 4. In step 1, Pt nanoparticles were encapsulated by adding K_2_PtCl_4_ and PAMAM dendrimers in water in the presence of NaBH_4_ and stirred for 48 h. In step 2, polyaniline was attached to the carbon nanotube sidewalls in the presence of HCl and (NH_4_)_2_S_2_O_8_ to increase its conductivity. In the final step, poly(sodium-4-styrene sulfonate) was dissolved in a NaCl solution. PANI-CNTs were added to this solution under constant stirring. Then, a Pt-encapsulated dendrimer solution was added. Further stirring was continued. Then, it was centrifuged and washed with deionized water to obtain Pt-DEN-PANI-CNT nanocomposite.

##### Transition Metal Nanoparticle CNT Composites

Dopamine (DA) is a critical neurotransmitter associated with many neurological diseases. Its sensitive and selective detection is important for the early diagnosis of diseases related to abnormal levels of DA [53]. Molybdenum nanoparticles self-supported functionalized multi-walled carbon nanotubes (MoNPs/f-MWCNTs) based core-shell hybrid nanomaterial with an average diameter of 40–45 nm was fabricated for electrochemical DA detection. In this case, O-containing groups from the acid treatment of the MWCNTs served to tether Mo NPs. This composite has a large surface area and numerous electroactive sites. This composite detects DA as low as 0.01 µM quantitatively with its detection limit of 1.20 nM under CV.

Simultaneous detection using electrochemical sensors is emerging as a powerful assay for biomolecular analysis. The rapid and sensitive quantification of six biomolecules offers benefits to pathological research, clinical diagnosis, and pharmaceutical quality control. UA, Xanthine (XA), theophylline (TP), and theobromine (TB) are products emanating from purine metabolism. A lack of AA enhances the risk of scurvy. DA is related to Parkinson’s disease and attention-deficit hyperactivity disorder (ADHD). UA is reported to be a sign of hyperuricemia known to progress into gout. TB, TP, and XA are produced from a Xanthine-based nucleoside from various metabolic pathways. UA is the final oxidized product of the purine catabolism pathway. Since these biomolecules generally exist in urine samples, simultaneous selective detection of these molecules is necessary. Patel et al. reported on the synthesis of a novel nanocomposite of titanium dioxide nanorods with multi-walled carbon nanotubes (TiO_2_ NRs-MWCNTs) using a solvothermal method [54]. The TiO_2_ NRs-MWCNTs/GCE as a working electrode detected UA, XA, TP, and TB selectively in the presence of AA and DA. Simultaneous quantification of these six molecules was studied using DPV in a wide potential window ranging from −0.3 to 1.6 V vs. Ag/AgCl at pH 4.0. This study observed simultaneous well-separated anodic peaks at 0.13, 0.35, 0.50, 0.85, 1.10, and 1.28 V for AA, DA, UA, XA, TP, and TB, respectively, using TiO_2_ NRs-MWCNTs/GCE. All six calibration curves of these biomolecules have two segments of a linear relationship, showing a slope for low concentrations and a slope for higher concentrations. A possible reason for a large redox-active area was available on the electrode surface at lower concentrations of the molecules. On the other hand, accessible redox-active areas rapidly saturated at greater concentrations of the biomolecules, resulting in decreased sensitivity in the slope of the second linear segments in their calibration curves. Therefore, concentration breakpoints were seen for all of these analytes. This sensor showed a linear range of AA from 1.5 to 51.0 µM and 51.0 to 191.0 µM with an LOD of 0.51 µM; DA from 0.45 to 30.0 µM and 30.0 to 147.0 µM with an LOD of 0.06 µM; UA from 0.40 to 61.0 µM and 61.0 to 537.0 µM with an LOD of 0.05 µM; XA from 0.5 to 97.0 µM and 97.0 to 586.0 µM with an LOD of 0.09 µM; TP from 1.0 to 203.0 µM and 203.0 to 891.0 µM with an LOD of 0.56 µM; TB from 1.5 to 368.0 µM and 368.0 to 1653.0 µM with an LOD of 0.75 µM. Selective quantification of these molecules at oxidation peak currents does not show any interference with other biomolecules.

**Scheme 5:**
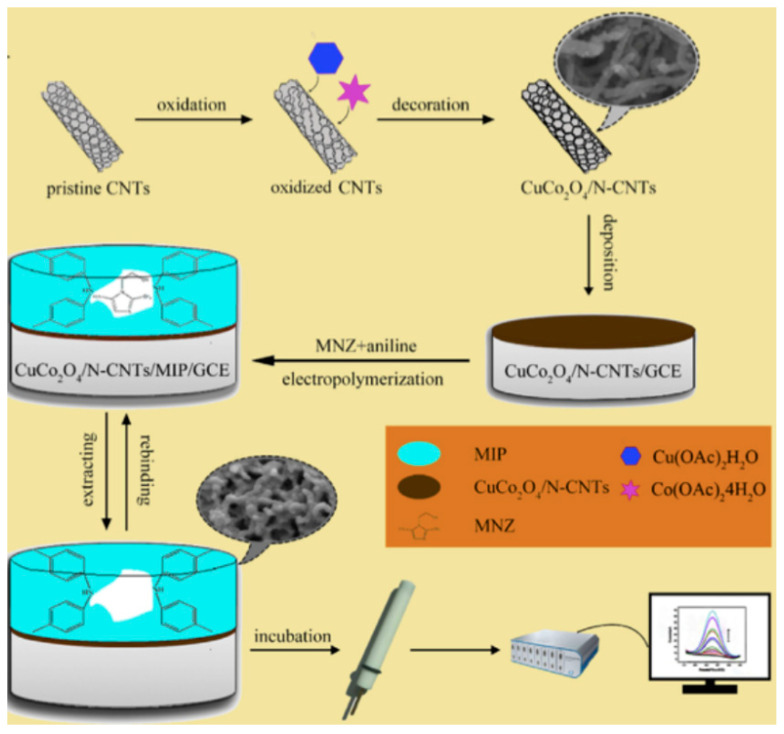
Schematic diagram for preparation of CuCo_2_O_4_/N-CNTs/MIP/GCE and electrochemical detection of metronidazole. Reproduced with permission from ref. [55]. Copyright 2019, Elsevier.

A CuCo_2_O_4_ nanoparticle modified with nitrogen-doped carbon nanotubes (CuCo_2_O_4_/N-CNTs) exhibits a high specific surface area (72.80 m^2^/g) and good electrical conductivity. N atoms served as the tethering points for the CuCo_2_O_4_ NPs. The CuCo_2_O_4_/N-CNTs loaded molecularly imprinted polymer (MIP) modified GCE was fabricated for fast and ultrasensitive detection of metronidazole (MNZ) using CV and DPV. Metronidazole is mainly used to treat and prevent amebic disease and echinococcosis caused by anaerobic bacteria. It belongs to the class of 5-nitroimidazole antibiotics. This sensor can selectively detect MNZ in presence of p-nitrophenol, 1-methylimidazole, L-phenylalanine, glycine, carbamide, dimetridazole, and 2-methyl-5-nitroimidazole in biological samples. These interfering analytes were chosen because they have the same chemical structures and sizes as the analyte [55]. In addition, various inorganic ions such as Na^+^, K^+^, Ca^2+^, Cl^−^, and SO_4_^2−^ were tested with this sensor to prove no significant interferences for the detection of MNZ. The experimental method is summarized in Scheme 5. Oxidation of MWCNTs takes place, and a one-step hydrothermal method is used to synthesize CuCo_2_O_2_/N-CNTs. This composite is used to modify the electrode to increase electrochemical signals. Then, the template molecule MNZ with the electrochemically active functional monomer aniline is electropolymerized on the surface of the CuCo_2_O_4_/N-CNT electrode.

Here, the specific recognition sites for MNZ were designed. With the removal of template molecules, cavities matching the MNZ spatial structure and binding site are achieved. As a result, MNZ is re-adsorbed by stacking and hydrogen bonding interaction. Electrochemical quantification of MNZ was determined by CV and DPV using this sensor.

GCEs modified by synthesized Fe_3_O_4_-MWCNTs-Ni NPs have been used to detect glucose with its low detection limit of 6.7 µM. Iron (II, III) oxide (Fe_3_O_4_) nanoparticles were in situ loaded on the surface of carboxylated multi-walled carbon nanotubes using chemical co-precipitation procedure. Prior to decorating Fe_3_O_4_-MWCNT through ultrasonication, the nickel nanoparticles were synthesized by reducing nickel chloride. This fabricated sensor was used to quantify glucose in honey and energy drinks [56].

Glycans that exist on glycoproteins play important roles in many biological processes such as cell growth and differentiation, cell-cell interactions, and protein folding. A change in glycan expression levels has often been associated with cancer development, progression, and metastasis. Glycans on cell surfaces are considered to be therapeutic targets or clinical biomarkers for the diagnosis of many harmful diseases. A thionine (Th)-bridged multi-walled carbon nanotube/gold nanoparticle (MWCNT/Th/Au NP) composite was fabricated by binding Au NPs to the surface of Th-coated MWCNTs, in which thionine acted as a linker to enable the negatively charged Au NPs to bind to the anionic MWCNT surface. Synthesized MWCNTs/Th/Au NP as a mediator/nanomaterial composite was used as an electrochemical biosensor for glycan assays on living cancer cells. Cell surface glycans, especially mannose, are closely associated with biological processes such as tumor growth and metastasis. Therefore, the study of mannose expression was significant for analyzing its role in cancer development. This sensor can detect mannose in the range of (0.015–0.045) µM. The biosensor was used to quantify 0.03328 µM mannose expression in QGY-7701 cells (at a concentration of (6 × 10^2^ cells per mL) [57]. In this case, the peak current obtained from mannose containing living cancer cells was concurrent with that obtained from standard mannose quantification. The sensor is selective towards mannose in the presence of other biological molecules such as amino acids and proteins. Table 1 shows a representative sampling of bioanalytes detectable using carbon nanotube-based composites. An additional array of CNT-based sensing composites are listed [58,59,60,61,62,63,64,65,66,67,68,69,70].

### 2.2. Graphene and Reduced Graphene Oxide

Graphene (G) is defined as a pristine two-dimensional sp^2^ hybridized carbon sheet. Since graphene was collected from bulk graphite by Geim and Novoselov in 2004, this 2D structure has had found widespread use [71]. It has a very large surface area, twice that of single-walled carbon nanotubes. It also possesses a tunable bandgap, a room temperature Hall effect, high mechanical strength, high thermal conductivity, and high electrical conductance. Graphene does not have metallic impurities that CNTs typically possess. Comparing CNTs with graphene, the production of graphene from graphite is more cost-effective and accessible [72].

Graphene oxide (GO) is a single graphitic monolayer with sp^2^ carbon atom and oxygenated aliphatic sp^3^ carbon atoms containing carbonyl, hydroxyl, epoxy, and carboxyl functional groups. The hydroxyl and epoxy groups lie below and above each graphene layer. The carboxylic groups generally appear at the edges of the layer. The presence of oxygen groups on the surface of GO offers a remarkable hydrophilic character, which can serve as tethering points for electrochemically active additives.

#### 2.2.1. Covalent Attachment of Functionalities to Graphene Oxide

Analogous to the CNT counterpart, catalytically active additives (metal NPs and metal oxide nanoparticles listed in Table 2) can be tethered using the O-containing moiety on the graphene surface. Whereas CNTs offer the O-containing tethering point via carboxylate/ester O atoms, GO does so via functionalization with –CH_2_OH terminated regioregular poly(3-hexylthiophene) (P3HT); this structure appears from the formation of ester bonds with the carboxyl groups of GO nanoplatelets. GO is grafted to polymeric chains with reactive species like hydroxyls and amine, i.e., poly(ethylene glycol), polyallylamine, poly(vinyl alcohol). Oligothiophenes are well-known conjugated polymeric materials with potential use in various optoelectronic applications: amine-terminated oligothiophenes are grafted on GO nanoplatelets through covalent amide bonds. The absence of carboxyl groups and the appearance of amide bonds have been studied in the FTIR of functionalized GO [73,74].

#### 2.2.2. Graphene and Reduced Graphene Oxide Noble Metal Composites

An array of graphene and graphene oxide based sensing composites are listed in Table 1 [75,76,77,78,79,80,81,82,83,84,85,86,87,88,89,90,91,92,93]. Phenylketonuria (PKU) is a condition resulting from a defect in the enzyme phenylalanine hydroxylase (PAH), preventing the metabolism of phenylalanine. PAH enzymes generally break down any excess unnecessary phenylalanine in the healthy body. Excess levels of phenylalanine are toxic, affecting the development and function of the brain. Diagnosing PKU at the early stages of life is important for preventing major health problems. PKU would result in brain damage if left untreated. Gold nanoparticles (AuNPs) decorated on the reduced graphene oxide sheets on the screen-printed electrode were fabricated to detect phenylketonuria-associated DNA mutations for the screening of PKU disease [75]. The composite was made by dispersing Au NPs and reduced graphene oxide (rGO) in a sonication bath followed by applying the colloidal suspension to a screen-printed carbon electrode (SPCE), which was then dried. A drop of single-stranded DNA was made to complete the working electrode for detecting nucleotide changes in the sample, signifying PKU. This sensor showed a linear dynamic range of 80 to 1200 fM of PAH DNA target concentrations with the detection limit of 21.3 fM using DPV.

#### 2.2.3. Graphene Oxide and Reduced Graphene Oxide Transition Metal Composites

Using transition metal oxide NPs to develop non-enzymatic quantification has gained traction due to ease of synthesis, cost-effective preparation, and low sensitivity to humidity and temperature. Foroughi et al. reported on graphene-modified CuO nanoparticles fabricated by microwave-assisted synthesis [76]. The material composite was placed on a glassy carbon electrode (GCE) to quantify glucose both at pH 13 and pH 7.4 by CV and CA. A linear analytical range from 5 mM to 14 mM glucose was detected with a limit of detection of 5 µM and a sensitivity of 37.62 µA∙mM^–1^∙cm^–2^ at the working potential of −0.03 V at pH 7.4 and scan rate of 50 mV∙s^−1^. However, a wide dynamic linear range of glucose from 0.21 µM to 12 mM was observed with an LOD of 0.21 µM using the same sensor at pH 13.

Raloxifene is an important chemotherapy drug used for cancer due to its antimetabolic and cytotoxic properties. It is used for clinical therapy as an antitumor and anthracycline antibiotic derivative of an anticancer drug. Raloxifene is very likely to communicate with DNA and can inhibit tumor cell proliferation. An overdose of the anticancer drugs could be harmful to biological functions, and the determination of raloxifene concentrations in human biological fluids is beneficial in the optimization of anticancer drug doses in chemotherapy treatments. Ultrasound-assisted synthesis of neodymium sesquioxide nanoparticles (Nd_2_O_5_ NPs) decorated GO nanocomposite under ultrasonic probe has also been reported for the detection of raloxifene [77]. The GCE modified with this composite exhibits excellent electrochemical sensing performance towards the anticancer drug raloxifene under CV and CA. This composite can detect raloxifene in a wide linear range from 0.030–472.3 µM with an LOD of 18.43 nM.

Graphene foam-like three-dimensional (3D) porous carbon/Ni nanoparticle (Ni NPs) nanocomposites were fabricated to detect glucose. Ni NPs were electrostatically attached to the graphene. Pomelo peel was used as a novel supporting material to load a large number of aqueous Ni^2+^ by a simple immersion method to form pomelo peel/Ni^2+^ which was then carbonized to prepare the graphene foam-like 3D porous carbon/Ni NPs nanocomposite (3D PPPC/Ni NPs). This nanocomposite has a wide linear range (15.84 µM–6.48 mM) of glucose detection with an LOD of 4.8 µM employing CV [78].

**Table 2:** Graphene based biosensors.

Sensor	Technique	Analyte	LOD	Reference
ssProbed modified SPCE/rGO/Au NP	DPV	PAH mutation	21.3 fM	[75]
CuO/Graphene	CA	Glucose	5.04 µM	[76]
Nd_2_O_5_ NPs/GO	CV	Raloxifene	18.43 nM	[77]
CNT/GO/Fe_3_O_4_	DPV	Diclofenac (DCF)	33 pM	[79]
CuSe/rGO	LSV	Eugenol	0.41 µg/kg	[80]
Pd-Mn/rGO	CA	Glucose	1.25 µM	[81]
RGO/Fe_3_O_4_	SWV	Melatonin	8.4 nM	[82]
RGO/ZrO_2_/Co_3_O_4_	CV, DPV	Gallic acid	1.56 nM	[83]
RGO/Au NPs/SPE	CV, DPV	Ascorbic acid	1.04 µM	[84]
RGO/GCE	CV, DPV	Resveratrol	0.2 µM	[85]
TiO_2_/rGO	CV, SWV	Oleuropein, Hydroxytyrosol	0.57 nM	[86]
NF/ERGO/GCE	DPV	Tocopherol	0.06 µM	[87]
RGO/GCE	CV, DPV	Curcumin	0.9 pM	[88]
GO-CMF/PdSPs	CV	Dopamine	23 nM	[89]
RGO/SnO_2_-Co_3_O_4_	CV, SWV	Melatonin	4.1 nM	[90]
RGO/CuO	CV, CA	Flutamide	0.001 µM	[91]
SPCE/AuNP/GO	LSV, EIS	Alkaline phosphatase	9.1 U/L	[92]
Gd_2_O_3_ NPs@rGO	CV, CA	H_2_O_2_	1.57 nM	[93]

A non-steroidal anti-inflammatory drug is diclofenac (2-(2-(2,6-dichlorophenyl amino)phenyl) acetic acid, DCF). A novel label-free electrochemical biosensor showed binding affinities with DCF ranging from 100–1300 pM with a detection limit of 33 pM. Such drugs are used to relieve pain and inflammation due to their analgesic and antipyretic properties. Diclofenac is used as an analgesic for postoperative conditions, such as rheumatoid arthritis, muscle pain, musculoskeletal disorders, and post-trauma inflammation. This drug and the metabolites formed in the human body enter the wastewater distribution systems via wastewater treatment plants, in which DCF is poorly eliminated. Azadbakht et al. reported on a biosensor fabricated with the acid-oxidized CNTs, GO, and Fe_3_O_4_ magnetic nanomaterials [79]. Sheets of CNT and GO were intertwined by ultrafine Fe_3_O_4_ nanoparticles forming 3D architectures. An animated detection probe (aptamer) was surface-confined on the CNT/GO/Fe_3_O_4_ surface through the covalent amide bonds formed by the carboxyl groups on the CNT/GO and the amino groups on the oligonucleotides at the 5′ end. The fabricated folding-based electrochemical sensor was used to detect target molecules utilizing structure-switching aptamers in the DPV setup. Signal was obtained from changes in electron transfer efficiency upon target-induced changes in the conformation of the aptamer probe. The decrease in DPV current signal for the interaction of aptamer/CNT/GO/Fe_3_O_4_/GCE with DCF confirmed the DCF/aptamer complex formation at the sensing platform. Such complexes become stable at the sensing interfaces. In this case, the resistive layer insulated the conductive support and reduced the DPV current signals. DPV signal was not observed at the maximum concentration of 1300 pM as the coverage of DFC at this concentration on the modified electrode reached a maximum limit. Such changes were easily quantified using the DPV technique.

Eugenol (C_10_H_12_O_2_) is a flavor compound in cloves, cinnamon, nutmeg, basil, and bay leaf. It is also an additive to cosmetics, and pharmaceutical products, and toothpaste as flavor. It may cause damage to the liver. An overdose of it causes a wide range of symptoms, from blood in the patient’s urine to nausea, convulsions, or a fast heartbeat. Therefore, it is necessary to fabricate new and rapid analytical techniques to determine eugenol in different real samples such as clove, cinnamon, and toothpaste. A modified glassy carbon electrode (CuSe/rGO/GCE) was used for various samples’ sensitive and selective voltammetric determination of eugenol. The reduced graphene oxide was decorated with copper selenide (CuSe/rGO), using supercritical carbon dioxide (Sc-CO_2_). It has gas-like diffusivity, extremely low viscosity, and excellent penetration ability, which helps to reduce GO sheets in order to disperse CuSe nanoparticles in between. Graphene is also used to serve as pores for encapsulating, thereby preventing the agglomeration of catalytically active NPs. The synthesized composite material, CuSe/rGO, was used to modify the GCEs. This sensor has a linear dynamic range between 1µg/kg to 82 µg/kg with an LOD of 0.41 µg/kg and recoveries between 88.5% and 94.8% [80].

**Scheme 6:**
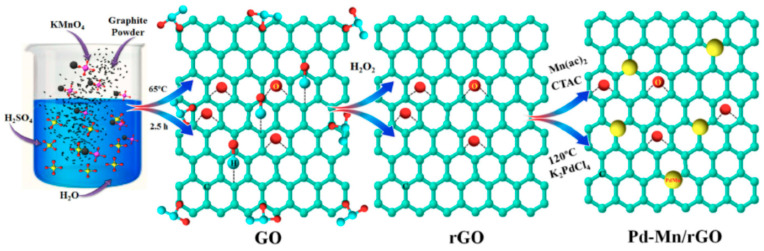
Schematic diagram presenting the systematic fabrication of Pd-Mn/rGO. Reproduced with permission from ref. [81]. Copyright 2020, John Wiley & Sons.

Palladium-manganese alloy nanoparticles supported on reduced graphene oxide (Pd-Mn/rGO) have been synthesized by a simple reduction protocol, important for applications in glucose sensing. The electrochemical activity and sensing features of this material towards glucose detection were studied amperometrically at a working voltage of −0.100 V. The increased electrochemical efficacy of Pd-Mn/rGO electrocatalyst is credited to the amount of electrocatalytic active sites that appears during Pd-Mn alloying and the electron transport ability of rGO that enhances the electron shuttling phenomenon between the electrode materials and targeted analyte. Monitoring glucose levels is important in order to reduce the danger of diabetes mellitus, a chronic progressive metabolic syndrome initiated by the absolute lack of insulin [81]. The LOD for glucose using this composite is 1.25 µM. Scheme 6 describes the synthesis method of Pd-Mn rGO composite. Within the graphene sheet structure, pores were available to entrain the electrocatalytically active Pd-Mn. Synthesis of GO was done using modified Hummer’s method. In this process, graphite powder was mixed with concentrated H_2_SO_4_ and magnetically stirred at 0 °C. KMnO_4_ was gradually introduced to the reaction mixture with continued stirring for 2 h in an oil bath. Distilled water was added slowly and H_2_O_2_ (30%) was also added slowly at room temperature. Finally, it was suction filtered with a HCl solution and washed several times with DI water; rGO was finally obtained as a dry product under vacuum. A modified solvothermal process was used as a reduction approach for depositing a nanoalloy of Pd-Mn on the surface of rGO. In this process, cetyltrimethylammonium chloride and rGO were dispersed in a water/ethylene glycol mixture and sonicated. In the second step, AA, K_2_PdCl_4_, Mn(CH_3_COO)_2_ were mixed into the solution. The whole reaction mixture was transferred into a Teflon bottle and heated in a hot air oven. Finally, the reaction mixture was cooled to room temperature. The Pd-Mn/rGO composite was collected using centrifuging, washing, and drying. An additional array of graphene-based sensing composites in Table 2 are listed [83,84,85,86,87,88,89,90,91,92,93].

Mahato et al. studied label-free EIS for alkaline phosphatase (ALP) detection, an indicator of cancer relapse and the onset of hepatitis C using AuNPs, electrochemically engineered Au-nano-dendroids, and GO nanocomposite [92]. These nanomaterials were sequentially deposited onto the screen-printed carbon electrode (SPCE) and antibodies against ALP (anti-ALP) were immobilized using a carbodiimide bioconjugation process. The designed biosensor (SPCE/AuNPs/Au-nano-dendroids/GO/anti-ALP) was evaluated using EIS with the linear dynamic range and the limit of detection of 100–1000 U/L and 9.10 (±0.12) U/L, respectively. Alkaline phosphatase is a phosphate-cleaving enzyme found in serum, saliva, and other bodily secretions and takes part in many vital physiological functions in biological systems. The sensor was able to quantify ALP in clinical serum samples under LSV, where the level was found to be 83.15 U/L. The physiological range of serum ALP in adults is 20–140 U/L in the case of a normal average adult, which increased in cases for infants, children, and pregnant women. The liver and bone associated disorders are related to ALP clinical ranges above 350 U/L. In addition, elevated serum ALP levels are associated with various cases of cancer relapse and at the onset of hepatitis C. Differently modified graphene and GO sensors for their various quantification of analytes with LODs are summarized in Table 2.

### 2.3. Carbon Dots

Carbon dots (CDs) are zero-dimensional carbon nanomaterials that have been polymerized. CDs have been extensively used as biosensors (Table 3). CDs are a subclass of nanoparticles, defined by a quasi-spherical morphology with a single unit with a characteristic size < 10 nm. They were discovered accidentally by Xu et al. in 2004 during the purification of single-walled carbon nanotubes [94]. Electroactive CDs are zero-dimensional carbon nanomaterials. These materials possess crucial electronic properties as possessed by quantum dots. They show low toxicity, stability, and biocompatibility for their application as electrochemical biosensors. CDs can also be classified as carbon quantum dots (CQDs) and graphene quantum dots (GQDs); we mention these alternative acronyms here as they are widely used in the literature. The CQDs and GQDs have a diameter range from 1 to 10 nm. GQDs consist of graphene layers of size less than 10 nm [95].

#### 2.3.1. Graphene Oxide and Reduced Graphene Oxide Transition Metal Composites

Carbon dots are mainly synthesized using top-down and bottom-up methods. Under top-down approaches, CDs are prepared by cutting larger carbon structure materials. Arc discharge, laser ablation, electrochemical synthesis, nanometer etching, hydrothermal, solvothermal, and special oxidation cleavage fall under the top-down methods. Xu et al. identified a mixture of unknown fluorescent nanoparticles when purifying SWCNTs from an arc discharge shoot [94]. Carbon dots were later synthesized by laser ablation of a suspension of carbon powders in poly(ethylene glycol) solvent, which simultaneously acted as a surface passivation agent [94,119]. The obtained CDs which have adequate polymer chains on the surface have a strong blue emission.

The electrochemical synthesis processes are applied more widely than arc discharge and laser ablation methods because of their simple operation and readily available equipment. Electrochemical reactions under the applied voltage at the electrode lead the carbon electrode materials to be corroded and exfoliated to obtain CDs. Graphite rods, multi-walled carbon nanotubes, carbon fibers, graphite powders, etc., are used as carbon electrode materials. Sodium hydroxide, t-butyl-3-methylimidazolium tetrafluoroborate, tetrabutylammonium perchlorate, phosphate buffer solution, potassium persulphate, and ultrapure water are used as the electrolytes. The research work of alkali metal-graphite intercalation compounds offers an avenue to prepare CDs. The metal-graphite intercalation method was used to exfoliate and disintegrate the multi-walled carbon nanotubes or graphite flakes to obtain CDs. The hydrothermal strategy to cut graphene sheets into surface-functionalized CDs was first introduced by Pan et al. [120]. The graphene oxide was dissolved in DMF and pyrolyzed in a poly(tetrafluoroethylene) (Teflon)-lined autoclave to obtain CDs under a one-step solvothermal route. Acid oxidation and photo-Fenton reactions were applied to form CDs by oxidation cleavage. Carbon dots can be readily synthesized using a microwave-assisted method [121,122] and hydrothermal method [123]. A study of the structure of carbon dots and their properties is very important to understand the structure-property relationship in CDs. For example, synthesized black CDs and carbon nitride dots appear to be more functionalized to a greater degree and more disordered and amorphous than yellow-CDs [124]. A direct conjugation of black CDs and gel-like CDs takes place through an amidation reaction to display many unique properties on novel drug carriers [125]. Amphiphilic CDs have been synthesized through hydrothermal carbonization using saccharides (D-glucose, D-galactose, and D-lactose) as the precursors [126]. CDs were prepared using tryptophan and two different dopants: urea and 1, 2 ethylenediamine [127]. Carbon nitride quantum dots (CNQDs) were first synthesized using four different precursor sets involving urea, thiourea, selenourea, and formamide [128]. CDs fractions have been prepared using a convenient one-step solvothermal route from gel-like materials [129]. A facile one-pot synthesis of a carbon dot gel material has been achieved by Ji et al. in 2017 [130]. Gel-like CDs (G-CDs) have been prepared using a rapid one-step solvothermal approach with citric acid and 1, 2-ethylenediamine as the precursors [131].

Nitrogen-rich carbon dots (N-CDs) have been reported to be well dispersed in aqueous solutions in large concentrations. Fiuza et al. discussed the potential of N-CDs to have tunable isoelectric points based on varying colloidal stability, solution pH, and ionic strength [132]. This possibility opens the door for its use as effective electrochemical sensing composites to better control the deposition of catalytically active NPs on these carbon supports by attaching them to the surface using Coulombic attraction.

#### 2.3.2. Functionalization of Carbon Dots

**Scheme 7:**
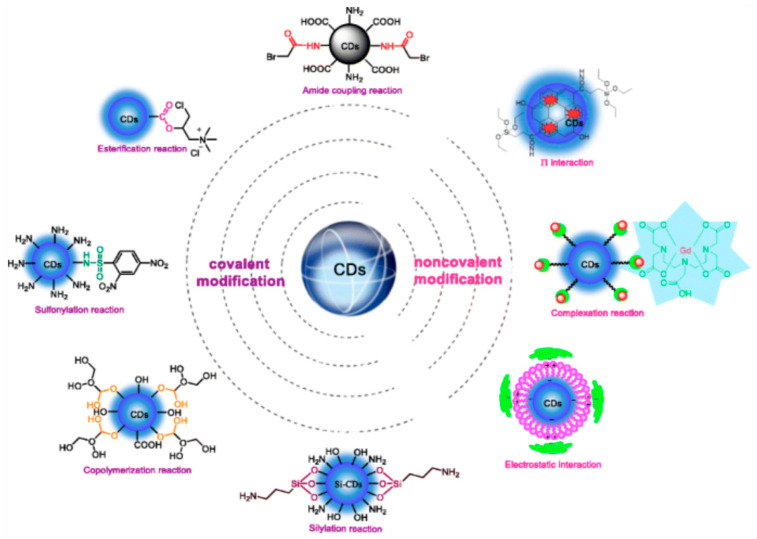
Various functionalizations for modification of carbon dots. Reproduced with permission from ref. [132]. Copyright 2018, Springer Nature.

Functionalized CDs, similar to CNTs, can have O-containing moeities serving as tethering points of electrocatalytically active additives (Table 3). CD-COOHs were modified with EDA via carbodiimide activated coupling reaction to yield CD-3 for the detection of citrate-silver nanoparticles based on the inner filter effect as shown in Scheme 7 [133]. The covalent modification through amide coupling reactions, silylation, and various reactions with sulfonylation, esterification, and copolymerization; and non-covalent modifications via complexation/chelation, interactions, and electrostatic interactions are summarized in Scheme 7. Such modification is vital for polymerization and can be used as capping agents for NPs, applicable for electrochemical sensing (*vide infra*, Section 2.3.3).

An amide coupling reaction is used to functionalize the CDs. The synthesis of CDs by treating urea, diethylenetriamine, PEI, and polyethylene glycol (PEG) as co-reaction reagents is generally rich in amino groups that affect high quantum yield and have very good biocompatibility. The presence of excessive amino groups on the surface of CDs shows low selectivity and water insolubility. Water insolubility of CDs indicates a good property for electrocatalysts. In addition, condensation of amino groups with acyl compounds using standard EDC/NHS-catalyzed reactions is applied as a surface modification to enhance the selectivity of CDs. This approach can be used for analyte detection and cell imaging [133,134,135,136]. The good stability and excellent electrochemical properties are shown by ferrocene (Fc). It has been applied to design PET-based organic molecular probes as an efficient electron donor.

Carbon quantum dots have been fabricated by microwave irradiation and were electropolymerized on GCE to sense ascorbic acid selectively. This analyte was studied by CV and DPV in the ranges of 0.11–3.0 mM and 4–12 mM, respectively. The limit of detection of AA was observed as low as 10 µM. Ascorbic acid or vitamin C plays an important role as an essential nutrient and antioxidant in the human body. The abnormal concentration of AA is related to different kinds of diseases, such as scurvy, mental disorder, cancer, AIDs, and digestive disorders. Also, as AA participates in key biological processes, e.g., cell division, iron absorption, acceleration of collagen synthesis, and melanogenesis inhibition, developing a sensor for assaying it is important. Regarding selectivity, the presence of DA and UA did not show any interference with the electrochemical detection of AA on the surface of the CQDs/GCE electrode. Moreover, there were no interferences with Na^+^, Cl^–^, Mg^2+^, SO_4_^–^, and glucose in samples at 100-fold concentration levels above that of the analyte using CA at +0.00 V [137].

#### 2.3.3. Biosensing Applications of Carbon Dots

##### Carbon Dot Transition Metal Oxide Composites

**Scheme 8:**
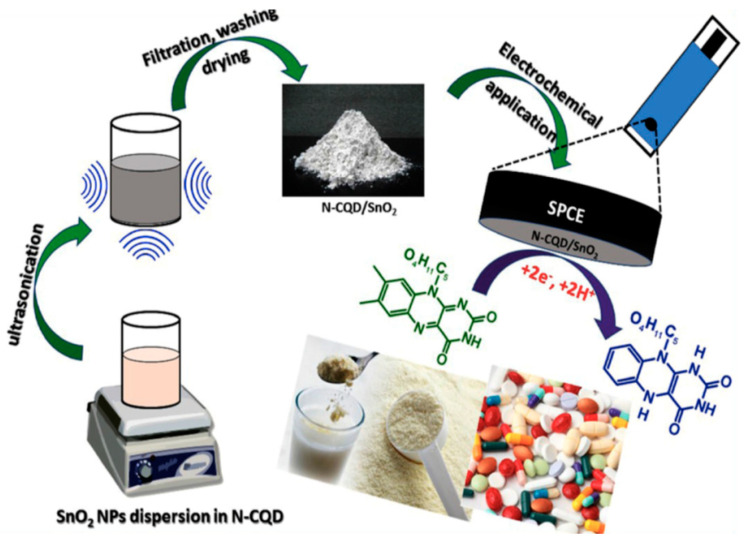
Schematic illustration for the sonochemical preparation of N-CQD/SnO_2_ nanocomposite and its electrochemical determination of RF in milk powder and tablets. Reproduced with permission from ref. [118]. Copyright 2019, Elsevier Ltd.

CDs with transition metals as electrocatalysts are important for enhancing the sensitivity of biological analytes. An array of CD-based sensing composites are listed in Table 3 [96,97,98,99,100,101,102,103,104,105,106,107,108,109,110,111,112,113,114,115,116,117,118]. Nitrogen-doped carbon quantum dots/SnO_2_ (N-CQD/SnO_2_) nanocomposite were fabricated using a sonochemical approach with N atoms serving as tethering points for SnO_2_ NPs. The prepared SnO_2_ NPs were mixed with synthesized N-CQD solution and ultrasonicated. The mixture was filtered to obtain N-CQD/SnO_2_ nanocomposite for quantification of riboflavin (RF) in tablets and milk powder, as shown in Scheme 8. This sensor was used to determine RF concentration using CV. This sensor shows a wide linear range (0.05–306) µM with an LOD of 8 nM RF. RF is a water-soluble vitamin (VB_2_). It assists in converting fats, proteins, and carbohydrates into energy in the human body. Riboflavin itself converts into coenzymes flavin mononucleotide (FMN) and flavin adenine dinucleotide (FAD). They are vital for tissue respiration. When there is a deficiency of RF, one may suffer from eye irritation, itching, sore tongue, and skin damage. Therefore, the sensitive determination of RF levels using N-CQD/SnO_2_ composite is necessary to monitor and diagnose such diseases [118].

Tryptophan is an essential amino acid for humans and herbivores, which is rarely available in vegetable products. It is a vital protein in the human diet and is responsible for maintaining a positive nitrogen balance. Tryptophan is also a precursor of the neurotransmitter serotonin. Tryptophan is of great importance in biochemical and pharmaceutical fields as it is a precursor molecule to hormones and neurotransmitters. It is also considered a possible cause of schizophrenia in people who cannot metabolize it properly. Under improper metabolization, it generates a waste product, a toxic analyte detected in the brain, causing hallucinations and delusions. Therefore, quantification of this amino acid is important for the detection of neuron-based diseases, such as schizophrenia, hallucinations, and delusions. A one-step electrodeposition method has been developed for the electrosynthesis of Fe_3_O_4_ magnetic nanoparticles/graphene quantum dots (Fe_3_O_4_-MNP-GQDs) on the surface of GCE. Applying this simple and effective deposition method, GQDs are effectively coated on the surface of electrodes. This sensor can quantify L-tryptophan (L-Trp) in the linear dynamic range of (0.08–150) µM with the LOD of 0.08 µM at physiological pH using DPV [99].

Cerebral Cu^2+^ is an essential trace element in the body, playing a vital role in various metabolic processes. Since it is a catalytic cofactor for many enzymes, including cytochrome C oxidase, tyrosinase, and superoxide dismutase, changes in cellular homeostasis of Cu^2+^ ions would result in cell death in addition to neurodegenerative (Alzheimer’s and Parkinson’s) diseases. Some amino compounds possess the capacity to identify copper ions selectively. With the modification of the surface of CDs, we can recognize Cu^2+^ ions selectively. In this electro-composite fabrication, N atoms served as the tethering point for the electrocatalytically active surface-attached additives. A specific recognition molecule AE-TPEA (N-(2-aminoethyl)-N, N’, N’-tris-(pyridine-2-yl-methyl) ethane-1, 2-diamine) was constructed for improving the selectivity of Cu^2+^ against other interfering metal ions. In this case, a functional group can coordinate with Cu^2+^ with high specificity to design a stable complex. Using silanization interactions, the CDs were tethered on (3-aminopropyl-) trimethoxysilane (APTMS) with a methoxy functional group. This was performed using electrochemical scanning to design a carbon-nitrogen linkage on the glassy carbon surface in advance. Finally, CDs functionalized with -OH and -COOH were conjugated via NHS and EDC as catalysts. This fabricated biosensor can selectively detect Cu^2+^ ions in a linear range of 1.0 to 60 µM with an LOD of 100 nM using differential pulse anodic stripping voltammetry (DPASV) due to specific recognition of the TPEA molecule [138]. Vancomycin (Van) is one of the glycopeptide antibiotics, which can be conjugated with CDs. The analyte is important for destroying harmful pathogens, such as *staphylococcus aureus* and methicillin-resistant *staphylococcus aureus* in the treatment of gram-positive bacterial infection.

UA (2, 6, 8-trihydroxy purine) is one of the fundamental waste products of nitrogenous base-like purine metabolism in humans. Various concentrations of UA levels studied have been found to relate to hyperuricemia, gout, Lesch-Nyhan disease, obesity, diabetes, high cholesterol, high blood pressure, and kidney disease. Quantifying UA in body fluids supports the diagnosis of these diseases. Hybrid nanocomposite (C-dots/Fe_3_O_4_ HCs) formation was obtained by supporting the adsorption of CDs over Fe_3_O_4_ NPs with amine-carboxyl interactions. The aggregation-free coverage of Fe_3_O_4_ NPs with a highly conductive layer of CDs as conductive centers is used to assist ultrafast electron transfer kinetics so that high signal sensitivity can be obtained. The enhancement of current signals and lower over-potential values enabled the generation of DC-amperometric (DC-AMP) sensors for uric acid (UA) with a linear working range of 0.010 to 0.014 µM and a signal sensitivity quantifiable up to 6.0 × 10^−9^ M. The enhancement of sensitivity results as a synergistic outcome of an active redox couple between Fe^2+^ and Fe^3+^, and a larger surface area of the Fe_3_O_4_ NPs were encapsulated with highly conductive CDs [97].

A carbon paste electrode (CPE) based on a novel nanocomposite of carbon dots/CuFe_2_O_4_ (C-dots/CuFe_2_O_4_) was fabricated by mixing pristine CuFe_2_O_4_ magnetic nanoparticles with synthesized CDs under ultrasonication for 1 h, followed by drying under vacuum at 80 °C for 12 h. This sensor is used for the simultaneous determination of rifampicin (RIF) and isoniazid (INZ) using CV and SWV, respectively. This sensor can detect RIF and NIZ in the ranges of 0.07 to 8.0 µM and 0.1 to 14.0 µM with the detection limits of 0.022 and 0.041 µM, respectively. This sensor holds great promise for fast, simple, and sensitive quantification of RIF and INZ in biological fluids and pharmaceutical samples. RIF and NIZ are classified as first-line anti-tuberculosis (anti-TB) drugs. These drugs treat *mycobacterium tuberculosis* (MTB) infection. Rifampicin, 3-[(4-methyl-1-piperazinyl)imino] methyl-rifamycin, also recognized as rifampin) is a semi-synthetic macrocyclic antibiotic used to treat tuberculosis (TB). Tuberculosis is a contagious bacterial infection caused by MTB, which affects the lungs. Rifampicin is also applied to fight *neisseria meningitidis* (a type of bacteria related to meningitis, a serious infection) infections in the nose or throat. This is also used to treat Hansen’s disease and other infections such as HIV. Isoniazid, pyridine-4-carboxylic acid hydrazide (also known as isonicotinyl hydrazine), is an antibiotic with significant bacterial activity in the initial phase of anti-TB therapy. The concentration of bacterial infection drugs is the key factor in controlling bacteria. Partial treatment of bacterial infections is common. The success or failure in therapeutic action is influenced by many factors such as the therapeutic regime, an interrupted drug supply, and/or chemical or physical interactions with other medications. However, an overdose of these drugs may lead to potential side effects, such as hepatotoxicity, allergic rashes, appetite loss, nausea, or immunological disorders. As the variations in bioavailability and pharmacokinetics of RIF and NIZ exist among people, it is necessary to fabricate therapeutic drug monitoring based on fast and reliable tools to quantify RIF and INZ concentrations [97].

Flutamide (FLU) and nitrofurantoin (NF) are anticancer and antibiotic drugs containing nitro groups, respectively. Flutamide, a non-steroidal anti-androgen drug is used to treat prostate cancer in men and to cure polycystic ovarian syndrome in women. Nitrofurantoin, an antibacterial agent is for urinary tract infections. It is used for pregnant women to treat infections in the urinary tract. However, the overdose of these drugs shows side effects, such as blood in urine, inflamed prostate, drowsiness, nausea, and liver malfunctions. In addition, the critical side effects of NF include inducing birth defects in embryos or fetuses. Moreover, due to the poor metabolism of NF and FLU, the metabolites are evacuated through urine and feces. The high electron-withdrawing nitro groups in FLU and NF resulted in poor biodegradation and kept the environment at risk. A simple microwave technique was applied to fabricate an N-CQDs/Co_3_O_4_ nanocomposite [100]. The electrocatalytic performance of the nanocomposite was enhanced with the presence of N-CQDs. Using the ultrasonication method, N-CQD/Co_3_O_4_ was hybridized with MWCNTs. The graphitic-conjugated structure of N-CQDs is responsible for tethering with MWCNTs for the formation of the hybrid N-CQD/Co_3_O_4_/MWCNTs. The synergistic effect between N-CQD, Co_3_O_4_, and MWCNT shows the high conductivity and high surface area of the hybrid nanocomposite. This sensor is used for the simultaneous determination of anticancer drugs flutamide (FLU) and nitrofurantoin (NF) using a GCE modified with N-CQDs/Co_3_O_4_/MWCNTs. This sensor shows a wide linear range of 0.05–590 µM for FLU and 0.05–1220 µM for NF with detection limits of 0.0169 µM and 0.044 µM, respectively. The selectivity of this sensor was studied using DPV in the presence of structurally relevant interfering molecules. The peaks of 100 µM FLU and NF were observed under DPV in presence of other analytes, such as 25-fold excess concentrations of nilutamide, (NLT), 4-nitroaniline (4-NA), 4-nitrophenol (4-NP), 4-nitrobenzene (4-NB), chloramphenicol (CAP), metronidazole (MTZ) and 50-fold excess concentrations of K^+^, Cu^2+^, and Br^-^ ions. The maximum amount of interfering molecules indicates the tolerance limit, responsible for 5% relative error for the quantitation of NF and FLU. This sensor shows excellent selectivity to the recognition of NF and FLU simultaneously using DPV in an aqueous solution in presence of other analytes.

##### Carbon Dots Modified with Noble Metals

One member of the aminoglycoside group purified from *streptomyces* is streptomycin (STR) analyte used in humans and veterinary applications to treat gram-negative infections. The presence of these residues in very little amounts (200 µg/kg) can trigger potential health hazards such as nephrotoxicity and ototoxicity. Electrochemical sensing systems based on GQDs functionalized with amine (-NH) and thiol (-SH) groups (GQDs-N-S) as promising new carbon-based nanomaterial were developed for the detection of STR. In this sensing system, gold nanoparticles (AuNPs) were deposited on the GCE surface. Then GQDs-N-S was coated on the Au NPs/GCE surface. Then, the silver nanoparticles (Ag NPs) were coated on the GQDs-N-S/Au NPs/GCE. This sensor detects streptomycin (STR) using CV and DPV. This sensor has an LOD of 0.0033 pg∙mL^−1^ with a linear range from 0.01 pg∙mL^−1^ to 812.21 pg∙mL^−1^. Amoxicillin, ciprofloxacin, and gentamicin are the interfering analytes for the detection of STR. However, this sensor shows a higher signal of STR compared to the above-mentioned other analytes, displaying excellent selectivity [98].

Attaching noble metals to carbon dots is important for rapid selective and sensitive detection of biomolecules, such as H_2_O_2_, uric acid, riboflavin, and quercetin (Table 3). Quercetin (3,3′,4′,5,7-pentahydroxy flavone), one of the abundant flavonoid molecules, is generally present in fruits and vegetables, especially in traditional Chinese herbs. It exhibits antioxidant, anti-inflammatory, anti-allergic, and antibacterial properties. It is clinically applied as a therapeutic medicine to reduce capillary permeability, lower blood pressure, and treat chronic bronchitis. On the other hand, with higher concentrations of quercetin in the biological body, adverse effects such as headache and kidney injury occur.

**Scheme 9:**
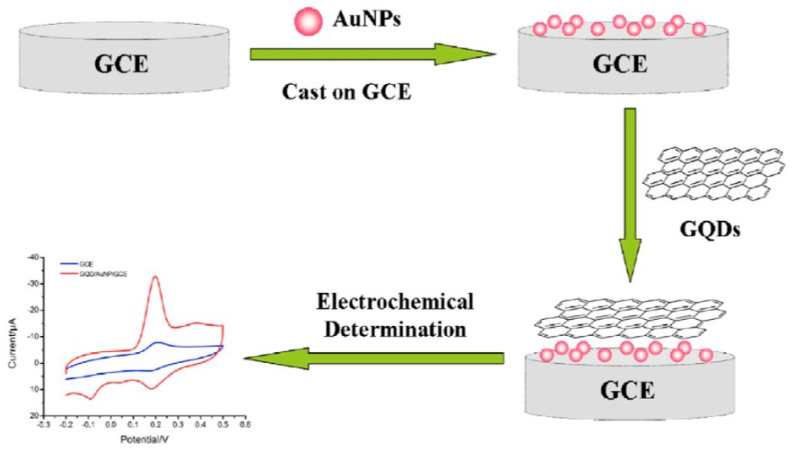
Schematic illustration of the fabrication of GQD/Au NP/GCE and the electrochemical determination of quercetin. Reproduced with permission from ref. [101]. Copyright 2016, John Wiley & Sons.

In an interesting development shown by Li et al., polymerized CDs can be used as a surface capping agent in the same manner as Nafion (vide supra, Section 2.3) [101]. The polymerized CD surface may be saturated with OH and -COO- moieties, which can attach to the NPs as it encapsulates the GCE surface. A drop of Au NPs of 10 µL was put onto the pretreated GCE surface to prepare the Au NPs/GCE. After drying in air, 10 µL GQDs (carbon dots in this application) was used to encapsulate Au NPs/GCE and dried under infrared lamps. The prepared GQDs/Au NP/GCE was rinsed many times to remove the residues. The nanocomposite of carbon dots and Au NP can highly accelerate the electron transfer rate and displays excellent synergistic electrochemical activity for the oxidation of quercetin. In this case (to demonstrate another application of the material), the carbon dots serve as capping agents similar to Nafion or chitosan, which is a departure from the tethering to O or N atom tethering point motif instead of serving as an NP support structure. The procedure for the fabrication of the modified electrodes and their electrochemical detection is depicted in Scheme 9. In preparing the electrode surface, Au NPs were drop-casted onto the GCE surface, followed by capping with polymerized C dots.

The Au NP/GCE was prepared by depositing 10 µL of Au NPs on the pretreated GCE. While drying in air, 10 µL GQDs were coated on the Au NP/GCE and dried under an infrared lamp. Finally, the prepared GQD/Au NP/GCE was rinsed with DI water to avoid residues. DPV was applied for the detection of quercetin. The oxidation peak current is proportional to the range of quercetin from 0.01 to 6.0 µM with an LOD of 2 nM. This sensor can successfully quantify quercetin sensitively and selectively in human plasma samples with (95.0–98.5)% recoveries.

Various potential interfering substances for the detection of quercetin in real samples were studied in the presence of a standard solution of 1.0 µM quercetin to assess the biosensor’s selectivity. At the maximum concentration of interfering substances, the tolerance limit caused about 5% relative error in the quantification of quercetin. A 50-fold ratio (relative to quercetin) of ascorbic acid, L-tryptophan, L-phenylalanine, L-cysteine; and a 100-fold ratio of oxalic acid, glucose, Mn^2+^, Ca^2+^; and a 500-fold ratio of Ca^2+^, Mg^2+^, Fe^3+^, Al^3+^, Zn^2+^ did not show any impact on the accurate concentration determination of quercetin in the medium. In addition, common flavonoids, such as apigenin, naringenin, and puerarin did not interfere with the concentration determination of quercetin due to weak signal responses from these compounds [101].

H_2_O_2_ is a byproduct of many reactions by most oxidase in mitochondria and produces reactive oxygen species (ROS). The proper amount of H_2_O_2_ and ROS plays a critical role in the function and signal transduction of living cells. Many tumor cells produce more H_2_O_2_ than of normal cells because of greater ROS production and have less ROS scavenging capacity due to abnormal tumor growth. The concentration of H_2_O_2_ released from living cancer cells is used as a valuable biomarker for many kinds of cancer for early-stage recognition. The combination of H_2_O_2′_s transient nature of H_2_O_2_ and matrix effects of cancer cells makes monitoring H_2_O_2_ molecules in cancer cells a challenge. Such obstacles need to be addressed by future researchers to advance biosensing technology. A new type of functionalized hollow-structured nanospheres (HNSs) based on Pd nanoparticles (NPs) decorated double-shell structured N-doped graphene quantum dots (NGQDs)/N-doped carbon (NC) HNSs, with ultrafine Pd NPs and nanozyme NGQDs having dual signal-amplifying nanoprobes were fabricated. Due to the synergistic effect of conductive HNS supports and catalytically active Pd NPs and NGQD in facilitating electron transfer, the NGQD/NC/Pd HNS hybrid material sensor shows the quantification of H_2_O_2_ using CV and CA. This sensor is able to assay H_2_O_2_ with an LOD of 20 nM and a dynamic range of 250 nM–1.4 mM level.

**Scheme 10:**
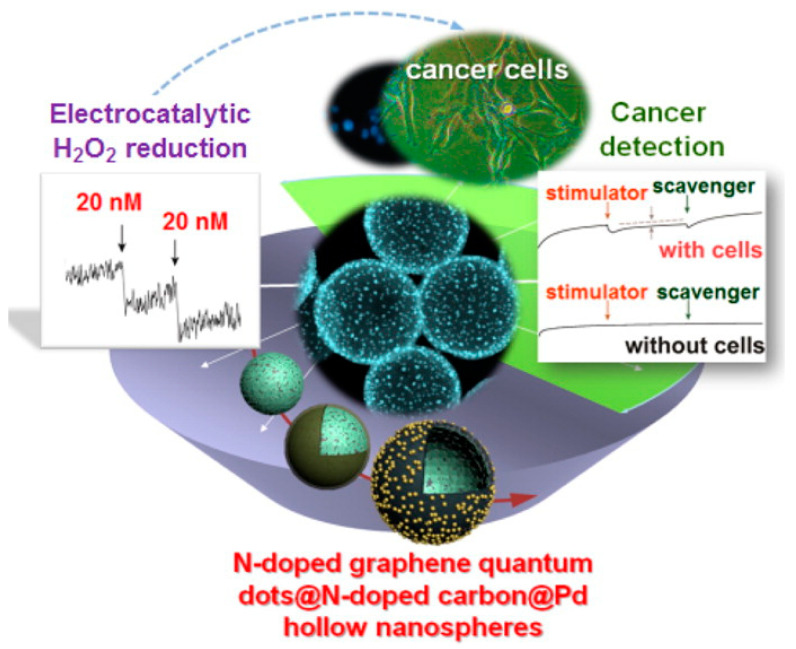
Electrochemical sensing performance in cancer detection. Reproduced with permission from ref. [102]. Copyright 2016, American Chemical Society.

H_2_O_2_ has been quantified in presence of interfering analytes, such as DA, AA, UA in PBS under CA using NGQD/NC/Pd HNS hybrid sensor. The good selectivity obtained from the favorable applied potential of 0.00 V for the amperometric study of H_2_O_2_ in which other interfering electroactive redox species do not show any response in physiological samples [102]. A large surface area, fast electron transfer rate, biocompatibility, and high electrocatalytic activity of the composite were exploited to achieve such superior sensitivity and selectivity. The detection limit of this composite is comparable to that of PB/ZnO/COOH-MWCNTs. Scheme 10 displays the ability of the NGQD/NC/Pd HNS hybrid sensor for analyzing H_2_O_2_, a cancer biomarker. The electrocatalytic H_2_O_2_ quantification in live cancer cells as low as 20 nM is the current extent of detection capability in this medium and in real-time conditions. The quantification of nanomolar levels of H_2_O_2_ excreted from various living cancer cells in conjunction with chemotherapy and radiotherapy has been achieved.

Hydrazine-modified graphene quantum dots (HM-GQDs) were prepared by refluxing GQDs with hydrazine hydrate. Such HM-GQDs were hybridized with gold nanoparticles (Au NPs) through the redox reaction between HM-GQDs and AuCl_4_^−^. This sensor has unique electrochemiluminescence (ECL) properties of HM-GQDs and easy self-assembly with some biomolecules of Au NPs, important for H_2_O_2_ assaying for cancer detection [102] HM-GQDs are also useful as a novel ECL immunosensor of carcinoembryonic antigen (CEA) as a model target analyte. It displays a linear response range of CEA between 0.02 and 80 ng∙mL^−1^ with an LOD of 0.01 ng∙mL^−1^ using a CV setup. Carcinoembryonic antigen is reported as a tumor marker in clinical tests [96].

## 3. Summary and Future Perspective

The development of CNM-based electrochemical sensors has yielded promising results for analyzing various kinds of diseases and drugs. Design elements of CNT-based electrochemical composites are transferable to graphene and CDs—for instance, O and N atoms as tethering points for binding catalytically active additives and NPs to the electroactive surface. Graphene has 2D pore structures that can encapsulate the NPs. Polymerized CDs have the added capability of functioning as capping agents for adsorbed GCE NPs. Indeed, CDs and graphene can function as effectively as CNTs electrocatalyst supports for biosensing applications. Additives containing more earth-rich metals, such as Zn, Cu, and Fe, show promise for achieving the same level of sensing performance as their precious-metal-based composite (e.g., Au, Ag, and Pt NPs) counterparts. The inclusion of earth-rich materials with CNMs will play a key role in advancing future technologies for performing sensing tasks more cost-effectively and in an environmentally friendly manner.

## Figures and Tables

**Table 1:** Detectable bioanalytes using carbon nanotube composites.

Sensor	Technique	Analyte	LOD	Reference
Pt/Ni(OH)_2_/MWCNT/GCE	CV	Nitrite	0.13 mM	[49]
SPCE-MWCNT/AuNPs	LSV	HER2-ECD	0.16 ng∙mL^–1^	[50]
MWCNTs/PEDT-Au	EIS, CV, SWV	Luteolin	0.2 nM	[51]
GO-COOH/MWCNT-COOH/PEI/Au	CV	Urinary 8-OHdG	7.06 nM	[52]
Mo NP/f-MWCNTs	CV	Dopamine	1.26 nM	[53]
CuCo_2_O_4_/N-MWCNT/GCE	CV	Metronidazole	0.48 nM	[55]
Fe_3_O_4_-MWCNT-Ni NPs	CV, CA	Glucose	6.7 µM	[56]
MWCNTs/Th/Au NP	DPV	Mannose	0.015 µM	[57]
GlcDH/PNb-SWNT/GCE	CA	Glucose	5.0 µM	[58]
AlcDH/PNb-SWNT/GCE	CA	Ethanol	50 µM	[59]
DSDH/PMG/MWCNT/GCE	CA	Sorbitol	100µM	[60]
PTBO/MWCNT/GCE	CA	NADH	0.5 µM	[61]
MWCNT/PMB/GCE	CA	H_2_O_2_	20.7 µM	[62]
PmalG/MWCNT/GCE	DPV	AA	0.23 µM	[63]
SWCNT/PTBO/GCE	CA	Nitrite	0.37 µM	[64]
PBG/MWCNT/CFE	DPV	DA	1.60 µM	[65]
PMB/MWCNT/GCE	CV	EP	69.6 µM	[66]
PmalG/MWCNT/GCE	DPV	UA	0.12 µM	[63]
EBNBH modified CNT-CPE	DPV	UA	15 µM	[67]
SWCNT mat grown on Si	CV	DA	1 µM	[68]
CNT paste with 2-PHC	SWV	Epinephrine	9 nM	[69]
SWCNT-inlaying ultrathin CPE	DPV	Xanthine	0.1 µM	[70]

Abbreviations: PMG = polymethylene green; PTBO = poly(toluidine blue O); PmalG = poly(malachite) green; PMB = poly(brilliant) green; EBNBH = ethanediylbis(nitriloethylidyne)]-bis-hydroquinone.

**Table 3:** Bioanalytes detected using carbon dot composites.

Sensor	Technique	Analyte	LOD	References
HM-GQD-AuNPs	CV, ECL	Carcinoembryonic antigen	0.01 ng∙mL^–1^	[96]
CDs/Fe_3_O_4_	DCAMP	Uric acid	0.006 µM	[97]
CDs/CuFe_2_O_4_/CPE	SWV	RIF, NIZ	0.022, 0.041 µM	[98]
AgNPs/CD-N-S/Au NPs	DPV	Streptomycin	0.036 pg∙mL^–1^	[99]
Fe_3_O_4_MNP-GQDs	DPV	L-tryptophan	0.08 µM	[100]
N-CDs/Co_3_O_4_/MWCNTs	DPV	Flu, NF	0.0169, 0.044 µM	[101]
AuNP/GQDs	DPV	Quercetin	2.0 nM	[102]
NGQDs/NC/Pd	CA	H_2_O_2_	20 nM	[103]
GQDs/2D-hBN/GCE	DPV	Serotonin	0.2 pM	[104]
Mag/NP/CQDs/SPE	DPV	NADH	20 nM	[105]
Β-CD-GQD/GCE	SWV	AA	0.49 µM	[106]
CQDs/Lac/GCE	CV	Epinephrine	83 nM	[107]
Β-CD@N-GQDs/Fc/GCE	DPV	Cholesterol	0.08 µM	[108]
CQDs/Cu_2_O/GCE	CA	Glu	6 µM	[109]
CQDs/MoS_2_/Mo foil	CV	DA	0.0090 µM	[110]
CQDs/SPE	DPV	DA	0.05 µM	[111]
Β-CD/CQDs/GCE	DPV	UA	0.01 µM	[112]
CQDs/GCE	CA	H_2_O_2_	300 nM	[113]
Ag NPs/CDs/GCE	CA, CV	H_2_O_2_	80 nM	[114]
NF/Hb/β-GQDs/CILE	DPV	H_2_O_2_	0.04 mM	[115]
GD-RuCl_3_/GCE	DPV	L-tyrosine	0.23 µM	[116]
Ppy/CDs@PB/GF	CV, CA	L-Cysteine	0.15 µM	[117]
N-CQD/SnO_2_/SPCE	DPV	Riboflavin	8 nM	[118]

## Data Availability

Not applicable.

## References

[1] Liu Z., Robinson J.T., Tabakman S.M., Yang K., Dai H. (2011). Carbon materials for drug delivery and cancer therapy. Mater. Today.

[2] Mendes R.G., Bachmatiuk A., Büchner B., Cuniberti G., Ruemmeli M.H. (2013). Carbon nanostructures as multi-functional drug delivery platforms. J. Mater. Chem. B.

[3] Francis A.P., Thiyagarajan D. (2018). Toxicity of carbon nanotubes: A review. Toxicol. Ind. Health.

[4] Mottier A., Mouchet F., Pinelli E., Gauthier L., Flahaut E. (2017). Environmental impact of engineered carbon nanoparticles: From releases to effects on the aquatic biota. Curr. Opin. Biotechnol..

[5] Avant B., Bouchard D., Chang X., Hsieh H.-S., Acrey B., Han Y., Spear J., Zeep R., Knightes C.A. (2019). Environmental fate of multiwalled carbon nanotubes and graphene oxide across diferent aquatic ecosystems. Nanoimpact.

[6] Kurapati R., Mukherjee S.P., Martin C., Bepete G., Vázquez E., Pénicaud A., Fadeel B., Bianco A. (2018). Degradation of single-layer and few-layer graphene by neutropil myeloperoxidase. Angew. Chem. Int. Ed. Engl..

[7] Srivastava I., Sar D., Mukherjee P., Schwartz-Duval A.S., Huang Z., Jaramillo C., Civantos A., Tripathi I., Allain J.P., Bhargava R. (2019). Enzyme-catalyzed biodegradation of carbon dots follows sequential oxidation in a time dependent manner. Nanoscale.

[8] Yang N., Chen X., Ren T., Zhang P., Yang D. (2015). Carbon nanotube based biosensors. Sens. Actuators B Chem..

[9] Radushkevich V., Lukayanovich V.M. (1952). The structure of carbon forming in thermal decomposition of carbon monoxide on an iron catalyst. Russ. J. Phys. Chem..

[10] Iijima S. (1991). Helical microtubules of graphitic carbon. Nature.

[11] Iijima S. (2002). Carbon nanotubes: Past, present and future. Phys. B Condens. Matter.

[12] Deb A.K., Chusuei C.C., Suzuki S. (2013). Aqueous Solution Surface Chemistry of Carbon Nanotubes. Physical and Chemical Properties of Carbon Nanotubes.

[13] Sahoo N.G., Cheng H.K.F., Li L., Chan S.H., Judeh Z., Zhao J. (2009). Specific functionalization of carbon nanotubes for advanced polymer nanocomposites. Adv. Funct. Mater..

[14] Moore E., Wang P.Y., Vogt A.P., Gibson C.T., Haridas V., Voelcker N.H. (2012). Clicking dendritic peptides onto single walled carbon nanotubes. RSC Adv..

[15] Liao W. (2013). Effects of multiwalled carbon nanotubes functionalization on the morphology of mechanical and thermal properties of carbon fiber/vinyl ester composites. ACS Appl. Mater..

[16] Yoonessi M., Lebron-Colon M., Scheiman D., Meador M.A. (2014). Carbon nanotube epoxy nanocomposites: The effects of interfacial modifcations on the dynamic mechanical properties of the nanocomposites. ACS Appl. Mater. Interfaces.

[17] Mammeri F., Teyssandier J., Darche-Dugares C., Debacker S., Le Bourhis E., Chehimi M.M. (2014). Carbon nanotube-poly(methyl methacrylate) hybrid films: Preparation using diazonium salt chemistry and mechanical properties. J. Colloid Interface Sci..

[18] Zhang W., Zhou Q., Li Q., Chen G.X. (2014). Controlled dielectric properties of polymer composites from coating multiwalled carbon nanotubes with octa-acrylate silesquioxane through Diels-Alder cycloaddition and atom transfer radical polymerization. Ind. Eng. Chem. Res..

[19] Najafi-Shoa S., Roghani-Mamaqani H., Salami-Kalajahi M., Azimi R. (2016). Incorporation of epoxy resin and carbon nanotube into silica/siloxane network for improving thermal properties. J. Mater. Sci..

[20] Abdollahi A., Roghani-Mamaqani H., Salami-Kalajah M., Mousavi A., Razavi B., Shahi S. (2018). Preparation of organic-inorganic hybrid nanocomposites from chemically modified epoxy and novolac resins and silica-attached carbon nanotubes by sol-gel process: Investigation of thermal degradation and stability. Prog. Org. Coat..

[21] Stobinski L. (2010). Multiwall carbon nanotubes purification and oxidation by nitric acid studied by the FTIR and electron spectroscopy methods. J. Alloys Compd..

[22] Shao Y., Yin G., Zhang J., Gao Y. (2006). Comparative investigation of the resistance to electrochemical oxidation of carbon black and carbon nanotubes in aqueous sulfuric acid solution. Electrochim. Acta.

[23] Jiang L.C., Zhang W.D. (2009). Electrodeposition of TiO_2_ nanoparticles on multiwalled carbon nanotube arrays for hydrogen peroxide sensing. Electroanalysis.

[24] Hu X., Zou C., Zou X. (2019). The formation of supramolecular carbon fiber via amidation reaction on the surface of amino single walled carbon nanotubes for selective adsorption organic pollutants. J. Colloid Interf. Sci..

[25] D’Arlas B.F., Goyanes S., Rubiolo G.H., Mondragon I., Corcuera M.A., Eceiza A. (2009). Surface modification of multiwalled carbon nanotubes via esterification using a biodegradable polyol. J. Nanosci. Nanotechnol..

[26] Iannazzo D., Piperno A., Ferlazzo A., Pistone A., Milone C., Lanza M., Cimino F., Speciale A., Trombetta D., Saija A. (2012). Functionalization of multiwalled carbon nanotubes with coumarin derivatives and their biological evaluation. Org. Biomol. Chem..

[27] Zhang C., Li F., Huang S., Li M., Guo T., Mo C., Pang X., Chen L., Li X. (2019). In-situ facile preparation of highly efficient Copper/Nickel bimetallic nanocatalyst on chemically grafted carbon nanotubes for non-enzymatic sensing of glucose. J. Colloid Interface Sci..

[28] Peng H., Alemany L.B., Margrave J.L., Khabashesku V.N. (2003). Sidewall carboxylic acid functionalization of single-walled carbon nanotubes. J. Am. Chem. Soc..

[29] Gu Y.J., Cheng J., Jin J., Cheng S.H., Wong W.T. (2011). Development and evaluation of pH-responsive single walled carbon nanotube-doxorubicin complexes in cancer cells. Int. J. Nanomed..

[30] Ensafi A.A., Zandi-Atashbar N., Rezaei B., Ghiaci M., Chermahini M.E., Moshiri P. (2016). Non-enzymatic glucose electrochemical sensor based on silver nanoparticle dcorated organic functionalized multiwall carbon nanotubes. RSC Adv..

[31] Xing Y., Li L., Chusuei C.C., Hull R.V. (2005). Sonochemical oxidation of multiwalled carbon nanotubes. Langmuir.

[32] Chusuei C.C., Wayu M., Marulanda J.M. (2011). Characterizing Functionalized Carbon Nanotubes for Improved Fabrication in Aqueous Solution Environments. Electronic Properties of Carbon Nanotubes/Book 5.

[33] Hull R.V., Li L., Xing Y., Chusuei C.C. (2006). Pt nanoparticle binding on functionalized multiwalled carbon nanotubes. Chem. Mater..

[34] Das S.C., Pandey R.R., Devkota T., Chusuei C.C. (2018). Raman spectroscopy as an assay to disentangle zinc oxide carbon nanotube composites for optimized uric acid detection. Chemosensors.

[35] Kader M.S., Chusuei C.C. (2020). A cobalt (II) oxide carbon nanotube composite to assay dopamine. Chemosensors.

[36] Zhao X., Aoki K.J., Chen J., Nishiumi T. (2014). Examination of the Gouy-Chapman theory for double layer capacitance in deionized latex suspensions. RSC Adv..

[37] Park J., Regalbuto J.R. (1995). A simple and accurate determination of oxide PZC and the strong buffering effect of oxide surfaces at incipient wetness. Colloid Interface Sci..

[38] McPhail M.R., Sells J.A., He Z., Chusuei C.C. (2009). Charging Nanowalls: Adjusting the Carbon Nanotube Isoelectric Point via Surface Chemical Functionalization. J. Phys. Chem. C.

[39] Pandey R.R., Guo Y., Gao Y., Chusuei C.C. (2019). A Prussian Blue ZnO carbon nanotube composite for chronoamperometrically assaying H_2_O_2_ in BT20 and 4T1 breast cancer cells. Anal. Chem..

[40] Gatabi M.P., Moghaddam H.M., Ghorbani M. (2016). Point of zero charge of maghemite decorated multiwalled carbon nanotubes fabricated by chemical precipitation method. J. Mol. Liq..

[41] Manasa G., Bhakta A.K., Mekhalif Z., Mascarenhas R.J. (2019). Voltammetric study and rapid quantification of rescorcinol in hair dye and biological samples using ultrasensitive maghemite/MWCNT modified cargon paste electrode. Electroanalysis.

[42] Deb A.K., Das S.C., Saha A., Wayu M.B., Marksberry M.H., Baltz R.J., Chusuei C.C. (2016). Ascorbic acid, acetaminophen, and hydrogen peroxide detection using a dendrimer-encapsulated Pt nanoparticle carbon nanotube composite. J. Appl. Electrochem..

[43] Wang X., Wu M., Tang W., Zhu Y., Wang L., Wang Q., He P. (2013). Simulataneous electrochemical determination of ascorbic acid, dopamine, and uric acid using a Palladium nanoparticles/graphene/chitosan modified electrode. J. Electroanal. Chem..

[44] Heitner-Wirguin C. (1996). Recent advances in perfluorinated ionomer membranes: Structures, properties, and applications. J. Mem. Sci..

[45] Mauritz K.A., Moore R.B. (2004). State of understanding of Nafion. Chem. Rev..

[46] Poletti F., Favarette L., Kovtun A., Treossi E., Corticelli F., Gazzano M., Palermo V., Zanardi C., Melucci M. (2020). Electrochemical sensing of glucose by chitosan modified graphene oxide. J. Phys. Mater..

[47] Kuppens I., Van Maanen M., Rosing H., Schellens J., Beijen J. (2005). Quantitative analysis of docetaxel in human plasma using liquid chromatography coupled with tandem mass spectrometry. Biomed. Chromatogr..

[48] Najari S., Bagheri H., Monsef-Khoshhesab Z., Hajian A., Afkhami A. (2018). Electrochemical sensor based on gold nanoparticle-multiwall carbon nanotube nanocomposite for the sensitive determination of docetaxel as an anticancer drug. Ionics.

[49] Sheng Q., Liu D., Zheng J. (2017). A nonenzymatic electrochemical nitrite sensor based on Pt nanoparticles loaded Ni(OH)_2_/multiwalled carbon nanotube composites. J. Electroanal. Chem..

[50] Freitas M., Nouws H.P.A., Delerue-Matos C. (2019). Electrochemical sensing platforms for HER2-ECD breast cancer biomarker detection. Electroanalysis.

[51] Cheng W., Zeng P., Ma C., Peng H., Yang J., Huang J., Zhang M., Cheng F. (2020). Electrochemical sensor for sensitive detection of luteolin based on multi-walled carbon nanotubes/poly(3,4-ethylenedioxythiophene)-gold nanocomposites. New J. Chem..

[52] Yi J., Qiao J., Wang Y., Gao K., Zhao R., Meng X. (2019). Electrochemical sensor platform for 8-hydroxy-2′-deoxyguanosine detection based on carboxyl-functionalized carbon-allotropic nanomaterials wrapped gold nanoparticles modified electrode. Int. J. Electrochem. Sci..

[53] Keerthi M., Boopathy G., Chen S.M., Chen T.W., Lou B.S. (2019). A core-shell molybdenum nanoparticles entrapped f-MWCNTs hybrid nanostructured material based non-enzymatic biosensor for electrochemical detection of dopamine neurotransmitter in biological samples. Sci. Rep..

[54] Patel B.R., Imran S., Ye W., Weng H., Noroozifar M. (2020). Simultaneous voltammetric detection of six molecules using a nanocomposite of titanium dioxide nanorods with multi-walled carbon nanotubes. Electrochim. Acta.

[55] Wang Y., Yao L., Liu X., Cheng J., Liu W., Liu T., Sun M., Zhao L., Ding F., Lu Z. (2019). CuCO_2_O_4_/N-doped CNTs with molecularly imprinted polymer for electrochemical sensor: Preparation, characterization, and detection of metronidazole. Biosens. Bioelectron..

[56] Nontawong N., Amatatongchai M., Jarujamrus P., Tamuang S., Chairam S. (2017). Non-enzymatic glucose sensors for sensitive amperometric detection based on simple method of nickel nanoparticles decorated on magnetite carbon nanotubes modified glassy carbon electrodes. Int. J. Electrochem. Sci..

[57] Zhang X., Huang C., Jiang Y., Shen J., Geng P., Zhang W., Huang Q. (2016). An electrochemical glycan biosensor based on a thionine-bridged multiwalled carbon nanotube/gold nanoparticle composite-modified electrode. RSC Adv..

[58] Du P., Wu P., Cai C. (2008). A glucose biosensor based on electrocatalytic oxidation of NADPH at single-walled carbon nanotubes functionalized with poly(Nile blue A). Electroanal. Chem..

[59] Du P., Liu S., Wu P., Cai C. (2007). Single-walled carbon nanotubes functionalized with poly(nile blue A) and their application to dehydrogenase-based biosensors. Electrochim. Acta.

[60] Wang Z., Etienne M., Pöller S., Schuhmann W., Kohring G.-W., Mamane V., Walcarius A. (2012). Dehydrogenase-based reagentless biosensors: Electrochemically assisted deposition of sol-gel thin films on functionalized carbon nanotubes. Electroanalysis.

[61] Zeng J., Wei W., Wu L., Liu X., Liu K., Li Y. (2006). Fabrication of poly(toluidine blue O)/carbon nanotube composite nanowires and its stable low-potential detection of NADH. J. Electroanal. Chem..

[62] Peña R.C., Bertotti M., Brett C.M.A. (2011). Methylene blue/multiwall carbon nanotube modified electrode for the amperometric determination of hydrogen peroxide. Electroanalysis.

[63] Raoof J.B., Ojani R., Baghayeri M. (2013). Fabrication of layer-by-layer deposited films containing carbon nanotubes and poly(malachite green) as a sensor for simultaneous determination of ascorbic acid, epinenphrine, and uric acid. Turk. J. Chem..

[64] Gligor D., Walcarius A. (2014). Glassy carbon electrode modified with a film of poly(toluidine blue O) and carbon nanotubes for nitrite detection. J. Solid State Electrochem..

[65] Ghica M.E., Winsterstellar Y., Brett C.M.A. (2013). Poly(brilliant green)/carbon nanotube-modified carbon film electrodes and application as sensors. J. Solid State Electrochem..

[66] Yogeswaran U., Chen S.-M. (2008). Multi-walled carbon nanotubes with poly(methene blue) composite film for the enhancement and separation of electroanalytical responses of catecholamine and ascorbic acid. Sensor Actuat. B..

[67] Mazloum-Ardakani M., Beitollahi H., Ganjipour B., Naeimi H., Nejati M. (2009). Electrochemical and catalytic investigations of dopamine and uric acid by modified carbon nanotube paste electrode. Bioelectrochemistry.

[68] Dumitrescu I., Edgeworth J.P., Unwin P.R., Macpherson J.V. (2009). Ultrathin carbon nanotube mat electrodes for enhanced amperometric detection. Adv. Mat..

[69] Beitollahi H., Karimi-Maleh H., Khabazzadeh H. (2008). Nanomolar and selective determination of epinephrine in the presence of nonepinephrine using carbon paste electrode modified with carbon nanotubes and novel 2-(4-oxo-3-phenyl-3, 4-dihydro-quinaZolinyl-)N’-phenyl-hydrazinecarbothioamide. Anal. Chem..

[70] Wang Z.H., Dong X.Y., Li J. (2008). An inlaying ultra-thin carbon paste electrode modified with functional single-wall carbon nanotubes for simultaneous determination of three purine derivatives. Sens. Actuators B-Chem..

[71] Novoselov K.S., Geim A.K., Morozov S.V., Jiang D., Zhang Y., Dobonos S.V., Grigorievo I.V., Frisov A.A. (2004). Electric field effect in atomically thin carbon film. Science.

[72] Pumero M., Ambrosi A., Bonanni A., Chng E.L.K., Poh H.L. (2010). Graphene for electrochemical sensing and biosensing. Trends Analyt. Chem..

[73] Georgakilas V., Otyepka M., Bourlinos A.B., Chandra V., Kim N., Kemp K.C., Hobza P., Zboril R., Kim K.S. (2012). Functionalization of graphene: Covalent and non-covalent approaches, derivatives, and applications. Chem. Rev..

[74] Vishwanathan P., Ramaraj R. (2019). Chapter 2-Functionalized graphene nanocomposite for electrochemical sensors. Graphene-Based Electrochemical Sensors for Biomolecules.

[75] Seifali S.M., Nasirizadeh N., Azimazadeh M. (2018). Nano-sensor based on reduced graphene oxide and gold nanoparticles, for detection of phenylketonuria-associated DNA mutation. IET Nanobiotechnol..

[76] Fouroughi F., Rhasepar M., Hadianfard M.J., Kim H. (2018). Microwave-assisted synthesis of graphene modified CuO nanoparticles for voltammetric enzyme-free sensing of glucose at biological pH values. Microchim. Acta.

[77] Chen T.-W., Merlin J.P., Chen S.-M., Anandraj S., Elshikh M.S., Tseng T.-W., Wang K., Qi D., Jiang J. (2020). Sonochemical synthesis and fabrication of neodyminum sesquioxide entrapped with graphene oxide based hierarchical nanocomposite for highly sensitive electrochemical sensor of anti-cancer (*Raloxifene*) drug. Ultrason. Sonochem..

[78] Wang L., Zhang Y., Yu J., He J., Yang H., Ye Y., Song Y. (2017). A green and simple strategy to prepare graphene foam-like three-dimensional porous carbon/Ni nanoparticles for glucose sensing. Sens. Actuat. B.

[79] Azadbakht A., Derikvandi Z. (2018). Aptamer-based sensor for diclofenac quantification using carbon nanotubes and graphene oxide decorated with magnetic nanomaterials. J. Iran. Chem. Soc..

[80] Murtada K., Moreno V., Ríos A., Zougagh M. (2019). Decoration of graphene oxide with copper selenide in supercritical carbon dioxide medium as a novel approach for electrochemical sensing of eugenol in various samples. J. Supercrit. Fluids.

[81] Waqas M., Lan J., Zhang X., Fan Y., Zhang P., Liu C., Jiang Z., Wang X., Zeng J., Chen W. (2020). Fabrication of non-enzymatic electrochemical glucose sensor based on Pd-Mn alloy nanoparticles supported on reduced graphene oxide. Electroanalysis.

[82] Bagheri H., Afkhami A., Hashemi P., Ghanei M. (2015). Simultaneous and sensitive determination of melatonin and dopamine with Fe3O_4_ nanoparticle-decorated reduced graphene oxide modified electrode. RSC Adv..

[83] Puangjan A., Chaiyasith S. (2016). An efficient ZrO_2_CO_3_O_4_ reduced graphene oxide nanocomposite electrochemical sensor for simultaneous determination of gallic acid, caffeic acid and protocatechuic acid natural antioxidants. Electrochim. Acta.

[84] Ji D., Liu Z., Liu L., Low S.S., Lu Y., Yu X., Zhu L., Li C., Liu Q. (2018). Smartphone-based integrated voltammetry system for simultaneous detection of ascorbic acid, dopamine, and uric acid with graphene and gold nanoparticles modified screen-printed electrodes. Biosens. Bioelectron..

[85] Liu L., Zhou Y., Kang Y., Huang H., Li C., Xu M., Ye B. (2017). Electrochemical evaluation of trans-resveratrol levels in red wine based on the interaction between resveratrol and graphene. Anal. Methods Chem..

[86] Yazar S., Kurtulbaş E., Ortaboy S., Atun G., Şahin S. (2019). Screening of the antioxidant properties of olive (*Olea europaea*) leaf extract by titanium based reduced graphene oxide electrode. Korean J. Chem. Eng..

[87] Filik H., Avan A.A., Aydar S., Çakar Ş. (2016). Determination of Tocopherol using reduced graphene oxide-Nafion hybrid-modified electrode in pharmaceutical capsules and vegetable oil samples. Food Anal. Methods.

[88] Dey N., Devasena T., Sivalingam T. (2018). A comparative evaluation of graphene oxide based materials for electrochemical non-enzymatic sensing of curcumin. Mater. Res. Express.

[89] Palanisamy S., Velusamy V., Ramaraj S., Chen S.-W., Yang T.C.K., Balu S., Banks C.E. (2019). Facile synthesis of cellulose microfibers supported palladium nanospindles on graphene oxide for selective detection of dopamine in pharmaceutical and biological samples. Mater. Sci. Eng. C.

[90] Zeinali H., Bagheri H., Monsef-Khoshhesab Z., Khoshsafar H., Hajian A. (2017). Nanomolar simultaneous determination of tryptophan and melatonin by a new ionic liquid carbon paste electrode modified with SnO_2_-CO_3_O_4_@rGO nanocompsite. Mater. Sci. Eng. C.

[91] Sakthinatan S., Kokulnathan T., Chen S.-M., Karthik R., Tamizhdurai P., Chiu T.-W., Shanthi K. (2019). Simple sonochemical synthesis of cupric oxide sphere decorated reduced graphene oxide composite for the electrochemical detection of flutamide drug in biological samples. J. Electrochem. Soc..

[92] Mahato K., Purohit B., Kumar A., Chandra P. (2020). Clinically comparable impedimetric immunosensor for serum alkaline phosphatase detection based on electrochemically engineered Au-nano-dendroids and graphene oxide nanocomposite. Biosens. Bioelectron..

[93] Manavalan S., Rajaji U., Chen S.-M., Chen T.-W., Ramalingam R.J., Maiyalagan T., Sathiyan A., Hao Q., Lei W. (2019). Microwave-assisted synthesis of gadolinium (II) oxide decorated reduced graphene oxide nanocomposite for detection of hydrogen peroxide in biological and clinical samples. J. Electroanal. Chem..

[94] Xu X., Ray R., Gu Y., Ploehn H.J., Gearheart L., Raker K., Scrivens W.A. (2004). Electrophoretic analysis and purification of fluorescent single-walled carbon nanotube fragments. J. Am. Chem. Soc..

[95] Kour R., Arya S., Young S.-J., Gupta V., Bandhoria P., Khosla A. (2020). Recent advances in carbon nanomaterials as electrochemical sensors. Electrochem. Soc..

[96] Dong Y., Wu H., Shang P., Zeng X., Chi Y. (2015). Immobilizing water-soluble graphene quantum dots with gold nanoparticles for low potential electrochemiluminescence immunosensor. Nanoscale.

[97] Abbas M.W., Soomro R.A., Kalwar N.H., Zahoor M., Avci A., Pehlivan E., Hallam K.R., Willander M. (2019). Carbon quantum dot coated Fe_3_O_4_ hybrid composites for sensitive electrochemical detection of uric acid. Microchem. J..

[98] Shiri S., Pajouheshpour N., Khossafar H., Amidi S., Bagheri H. (2017). An electrochemical sensor for the simulataneous determination of rifampicin and isoniazid using a C-dots@CuFe_2_O_4_ nanocomposite modified carbon paste electrode. New J. Chem..

[99] Roushani M., Ghanbari K., Hoseini S.J. (2018). Designing an electrochemical aptasensor based on immobilization of the aptamer onto nanocomposite for detection of the streptomycin antibiotic. Microchem. J..

[100] Hasanzadeh M., Karimzadeh A., Shadjou N. (2018). Magnetic graphene quantum dots as a functional nanomaterial towards voltammetric detection of L-tryptophan at physiological pH. J. Nanostruct..

[101] Muthusankar G., Devi R.K., Gopu G. (2020). Nitrogen-doped carbon quantum dots embedded CO_3_O_4_ with multiwalled carbon nanotubes: An efficient probe for the simultaneous determination of anti-cancer and antibiotic drugs. Biosens. Bioelectron..

[102] Li J., Qu J., Yang R., Qu L., Harrington P.D.B. (2016). A sensitive and selective electrochemical sensor based on graphene quantum dot/golds nanoparticle nanocomposite modified electrode for the determination of quercertin in biological samples. Electroanalysis.

[103] Xi J., Xie C., Zhang Y., Wang L., Xiao J., Duan X., Ren J. (2016). Pd nanoparticles decorated N-doped graphene quantum dots@N-doped carbon hollow nanospheres with high electrochemical sensing performance in cancer detection. ACS Appl. Mater. Interfaces.

[104] Yola M.L., Atar N. (2018). A novel detection approach for serotonin by graphene quantum dots/two dimensional (2D) hexagonal boron nitride nanosheets with molecularly imprinted polymer. Appl. Surf. Sci..

[105] Canevari T.C., Cincotto F.H., Gomes D., Landers R., Toma H.E. (2017). Magnetite nanoparticles bonded carbon quantum dots magnetically confined onto screen printed carbon electrodes and their performance as electrochemical sensor for NADH. Electroanalysis.

[106] Shadjou N., Hasanzadeh M., Talebi F., Marjani A.P. (2016). Integration of Beta-cyclodextrin in graphene quantum dot nano-structure and its application towards detection of vitamin C at physiological pH: A new electrochemical approach. Mater. Sci. Eng. C..

[107] Baluta S., Lesiak A., Cabaj J. (2018). Graphene quantum dots-based electrochemical biosensors for catecholamine neurotransmitters detection. Electroanalysis.

[108] Ganganboina A.B., Doong R. (2018). Functionalized N-doped graphene quantum dots for electrochemical determination of cholesterol through host-guest inclusion. Microchim. Acta..

[109] Maaoui H., Teodoresu F., Wang Q., Pan C.H., Addad A., Chtourou R., Szanerits S., Boukherroub R. (2016). Non-enzymatic glucose sensing using carbon quantum dots decorated with copper oxide nanoparticles. Sensors.

[110] Shang J., Zhao M., Qu H., Li H., Chen S. (2019). Fabrication of CQDs/MoS_2_/Mo foil for the improved electrochemical detection. Anal. Chim. Acta.

[111] Beitollah H., Dourandish Z., Ganjali M.R., Shakeri S. (2018). Voltammetric determination of dopamine in the presence of tyrosine using graphite screen-printed electrode modified with graphene quantum dots. Ionics.

[112] Chen J., He P., Bai H., He S., Zhang T., Zhang X., Dong F. (2017). Poly (Beta-cyclodextrin)/carbon quantum dots modified glassy carbon electrode: Preparation, characterization, and simultaneous determination of dopamine, uric acid and tryptophan. Sens. Actuators B.

[113] Sadhukhan M., Bhowmik T., Kundu M.K., Barman S. (2014). Facile synthesis of carbon quantum dots and thin graphene sheets for non-enzymatic sensing of hydrogen peroxide. RSC Adv..

[114] Jahanbakhshi M., Habibi B. (2016). A novel and facile synthesis of carbon quantum dots via salep hydrothermal treatment as the silver nanoparticles support: Application to electroanalytical determination of H_2_O_2_ in fetal bovine serum. Biosens. Bioelectron..

[115] Chen W., Weng W., Niu X., Li X., Men Y., Sun W., Li G., Dong L. (2018). Boron-doped graphene quantum dots modified electrode for electrochemistry and electrocatalysis of hemoglobin. J. Electroanal. Chem..

[116] Habibi E., Heidari H. (2016). Renewable surface carbon composite electrode bulk modified with GQD-RuC_13_ nano-composite for high sensitive detection of L-tyrosine. J. Electroanal. Chem..

[117] Wang L., Tricard S., Yue P., Zhao J., Fang J., Shen W. (2016). Polypyrrole and graphene carbon dots@Prussian Blue hybrid film on graphite felt electrodes: Application for amperometric determination of L-cysteine. Biosens. Bioelectron..

[118] Muthansankar G., Rajkumar C., Chen S.M., Karkuzhali R., Gopu G., Sangili A., Sengottuvelan N., Sankar R. (2019). Sonochemical driven simple preparation of nitrogen-doped carbon quantum dots/SnO_2_ nanocomposite: A novel electrocatalyst for sensitive voltammetric determination of riboflavin. Sens. Actuators B Chem..

[119] Xia C., Zhu S., Feng T., Yang M., Yang B. (2019). Evolution and synthesis of carbon dots: From carbon dots to carbonized polymer dots. Adv. Sci..

[120] Pan D., Zhang J., Li Z., Wu M. (2010). Hydrothermal route for cutting graphene sheets into blue-luminescent graphene quantum dots. Adv. Mater..

[121] Zhou Y., Zahran E.M., Quiroga B.A., Perez J., Mintz K.J., Peng Z., Liyanage P.Y., Pandey R.R., Chusuei C.C., Leblanc R.M. (2019). Size-dependent photocatalytic activity of carbon does with surface-state determined photoluminescence. Appl. Catal. B.

[122] Cilingir E.K., Seven E.S., Zhou Y., Walters B.M., Mintz K.J., Pandey R.R., Wikramanayake A.H., Chusuei C.C., Vanni S., Graham R.M. (2021). Metformin derived carbon dots: Highly biocompatible fluorescent nanomaterials as mitochondrial targeting and blood-brain barrier penetrating biomarkers. J. Colloid Interface Sci..

[123] Peng Z., Zhou Y., Ji C., Pardo J., Mintz K.J., Pandey R.R., Chusuei C.C., Graham R.M., Yan G., Leblanc R.M. (2020). Facile synthesis of ‘boron-doped’ carbon dots and their application in visible-light-driven photocatalytic degradation of organic dyes. Nanomaterials.

[124] Mintz K.J., Bartoli M., Rovere M., Zhou Y., Hettiarachchi S.D., Paudyal S., Chen J., Domena J.B., Liyanage P.Y., Sampson R. (2021). A deep investigation into the structure of carbon dots. Carbon.

[125] Zhou Y., Mintz K.J., Cheng L., Chen J., Ferreira B.C.L.B., Hettiarachchi S.D., Liyanage P.Y., Seven E.S., Miloserdov N., Pandey R.R. (2020). Direct conjugation of distinct carbon dots as Lego-like building blocks for the assembly of versatile drug nanocarriers. J. Colloid Interface Sci..

[126] Seven E.S., Sharma S.K., Meziane D., Zhou Y., Mintz K.J., Pandey R.R., Chusuei C.C., Leblanc R.M. (2019). Close-packed Langmuir monolayers of saccharide-based carbon dots at the air-subphase interface. Langmuir.

[127] Mintz J.K., Mercado G., Zhou Y., Ji Y., Hettiarachchi D.S., Liyanage Y.P., Pandey R.R., Chusuei C.C., Dallman J., Leblanc M.R. (2019). Tryptophan carbon dots and their ability to cross the blood-brain barrier. Colloids Surf. B.

[128] Liyanage P.Y., Geleroff D.L., Peng Z., Mintz K.J., Hettiarachch S.D., Pandey R.R., Chusuei C.C., Blackwelder P.L., Leblanc R.M. (2019). Carbon Nitride Dots: A Selective Bioimaging Nanomaterial. Bioconjug. Chem..

[129] Zhou Y., Liyanage P.Y., Geleroff D.L., Peng Z., Mintz K.J., Hettiarrachchi S.D., Pandey R.R., Chusuei C.C., Leblanc R.M. (2018). Photoluminescent carbon dots: A mixture of heterogeneous fractions. ChemPhysChem.

[130] Ji Y., Zhou Y., Waidely E., Desserre A., Marksberry M.H., Chusuei C.C., Dar A.A., Chat O.A., Li S., Leblanc R.M. (2017). Rheology of a carbon dot gel. Inorg. Chim. Acta.

[131] Zhou Y., Desserre A., Sharma S.K., Li S., Marksberry M.H., Chusuei C.C., Blackwelder P.L., Leblanc R.M. (2017). Gel-like carbon dots: Characterization and their potential applications. ChemPhysChem.

[132] Fiuza T., Gomide G., Campos A.F.C., Messina F., Depyrot J. (2019). On the colloidal stability of nitrogen-rich carbon nanodots aqueous dispersions. C J. Carbon Res..

[133] Yan F., Jiang Y., Sun X., Bai Z., Zhang Y., Zhou X. (2018). Surface modification and chemical functionalization of carbon dots: A review. Microchim. Acta.

[134] Yan F., Kong D., Luo Y., Ye Q., He J., Guo X., Chen L. (2016). Carbon dots serve as an effective probe for the quantitative determination and for intracellular imaging of mercury (II). Microchim. Acta.

[135] Yin J.Y., Liu H.J., Jiang S., Chen Y., Yao Y. (2013). Hyperbranched polymer functionalized carbon dots with multi stimuli-responsive property. ACS Macro Lett..

[136] Shi W., Li X., Ma H. (2012). A tunable ratiometric pH sensor based on carbon nanodots for the quantitative measurement of the intracellular pH of whole cells. Angew. Chem. Int. Ed. Eng..

[137] Wei Y., Zhang D., Fang Y., Wang H., Liu Y., Xu Z. (2019). Detection of ascorbic acid using green synthesized carbon quantum dots. J. Sens..

[138] Shao X., Gu H., Wang Z., Chai X., Tian Y., Shi G. (2013). Highly selective electrochemical strategy for monitoring of cerebral Cu^2+^ based on a carbon dot-TPEA hybridized surface. Anal. Chem..

